# Supplementary material for: BALSA: integrated secondary analysis for whole-genome and whole-exome sequencing, accelerated by GPU
Source: PeerJ. 2014 Jun 3;2:e421. doi: 10.7717/peerj.421 (PMC4060040; doi:10.7717/peerj.421)
Supplement: Supplemental Information 1 — Supplementary Methods and Appendix [file peerj-02-421-s001.docx]

**Department of Computer Science, The University of Hong Kong**

08

**Fall**

BALSA Supplementary Document

Version 1.1

Catalogue

1 Overview 5

1.1 Data Input 5

1.2 Data Output 5

1.3 Workflow 6

2 Resource Consumption 7

2.1 CPU 7

2.2 GPU 7

2.3 RAM 7

2.4 IO 7

2.5 Tweak 7

2.5.1 Reducing Linux swappiness 7

2.5.2 Using Transparent Huge Pages 8

2.6 Critical parameters in configuration file 8

3 Base Quality Score Recalibration 9

3.1 Workflow and descriptions 9

3.2 Reads sampling 9

3.3 Score Recalibration and Convergence Test 10

3.4 Example pre and post recalibration results 10

3.5 Future Work 11

4 Read Alignment and On-the-fly Pileup 12

4.1 Workflow and descriptions 12

4.2 Per Base Information 12

4.2.1 Weighted Count 12

4.2.2 Strand Count 13

4.2.3 Soft Clip Count 13

4.2.4 Indel Count and Insertion Pattern 13

4.3 Space-efficient Data Structures 13

4.4 Temporary files 17

5 De-duplication 19

5.1 Workflow and descriptions 19

5.2 Duplicate identification 20

5.3 Future Work 20

6 Indel Realignment 21

6.1 Rationale 21

6.2 Workflow 21

6.3 Realignment Windows and Regions 22

6.3.1 Realignment windows and triggering conditions 22

6.3.1.1 Condition 1 23

6.3.1.2 Condition 2 23

6.3.1.3 Condition 3 23

6.3.1.4 Condition 4 23

6.3.1.5 Condition 5 24

6.3.1.6 Condition 6 24

6.3.1.7 Condition 7 24

6.3.1.8 Condition 8 24

6.3.2 Realignment regions 24

6.4 Indel Patterns Extraction 25

6.4.1 Alignment, IndelDB, DP with modified scoring matrix 25

6.5 DP with modified scoring matrix 26

6.6 Modified References with Indel patterns 26

6.7 DP alignment on modified reference 27

6.7.1 Selecting reads for realignment. 27

6.7.2 DP realignment 27

6.8 Best alignment Selection 28

7 SNAPSHOT 30

8 Variants Calling 32

8.1 Workflow 32

8.2 16-Genotype Model 32

8.3 Variant classification using Random Forest 34

8.3.1 Features used for model training 35

8.3.2 Features used for selecting “false sites” 35

8.3.3 Model training details 35

8.4 Output 36

8.4.1 Variant Calling Format (VCF) 36

9 Somatic Calling 37

9.1 Workflow 37

9.2 Problem Formulation 37

9.3 Algorithm for detecting difference between normal and tumor 38

9.4 Output 39

10 CNV 40

10.1 Workflow and description 40

10.2 Identify candidate segments 41

10.3 Merge overlapping or adjacent candidates 41

10.4 Adjust segment boundaries 42

10.5 Remerge adjacent segments with similar log_2_ratio 42

10.6 Performance Evaluation 43

10.7 Output 44

1 Datasets 45

1.1 YH 100bp and 150bp paired-end read dataset 45

1.2 Simulated 40x 100bp paired-end read dataset 45

2 Commands 47

2.1 The BALSA pipeline (version r166) 47

2.1.1 Preparing databases for BALSA 47

2.1.2 From raw reads to variants 47

2.1.3 Filtration 48

2.1.4 Call somatic mutation 49

2.1.5 Call CNV 50

2.2 Running BWA 50

2.2.1 aln 50

2.2.2 mem 51

2.3 Running SOAP3-dp 51

2.4 Running the GATK pipeline and multiple (Ensemble) variant callers 51

2.4.1 Post-processing 51

2.4.2 Ensemble variant caller (Variant calling with 7 callers) 52

2.4.2.1 Atlas2 52

2.4.2.2 GATK UnifiedGenotyper and HaplotypeCaller 52

2.4.2.3 Freebayes 53

2.4.2.4 Mutect 53

2.4.2.5 SAMtools genotype calling 53

2.4.2.6 VarScan genotype calling 53

2.4.3 Integrating variant results from 7 callers 53

2.4.3.1 SNP integration 53

2.4.3.2 Indel integration 54

2.5 The Isaac pipeline 54

2.6 Running Mutect for somatic mutations 54

# Overview

## Data Input

There are two types of input data – BALSA-required indices and short reads.

**Indices** include 1) a 2way-BWT index (for alignment), 2) dbSNP database (for base quality score recalibration in section 3 and Random Forest based red in soorted suiltable ns in variant calling.tor and won'ter; 2) ted in lignment, realignment and statisvariant classification in section 8.3), 3) Indel database (hereafter referred to as IndelDB, for realignment in section 0) and 4) optionally, Gene region list (For realignment or using with exome sequencing mode).

dbSNP database and IndelDB contains known SNPs and Indel calls. The IndelDB can be 1000 Genomes and Mills Indel calls. A pre-processing tool will transform inputting VCF file into a binary dbSNP database and a single binary IndelDB database for BALSA’s input (Details introduced in Appendix 2.1).

**Short reads** are the short paired-end reads provided with the files in FASTQ format or BAM format.

In exome sequencing mode, Gene region list will limit modules including re-alignment and variant calling to work only within the gene regions.

## Data Output

BALSA supports variant output in both tab-delimited format and VCF format version 4.1. The VCF format can be converted from the tab-delimited format using a script.

Optional output includes a SNAPSHOT (5 files) storing the pileup details from short reads for downstream analysis. According to the specifications of the SNAPSHOT files introduced in section 7, users can improve the algorithms in the variant calling, somatic mutation calling and CNV calling modules introduced in this paper, or develop new algorithms to detect other types of mutations. A documented API to facilitate users to get different types of information from the SNAPSHOT is our future work.

## Workflow

# Resource Consumption

## CPU

BALSA by default uses 6 cores of CPU. Experiments carried out in this paper, if not explicitly mentioned, were using all 6 cores of an Intel® i7-3930k @3.2GHz with Hyper-Threading enabled.

## GPU

BALSA has been tested to be fully compatible with the following Nvidia GPU acceleration devices:

- Tesla C2050, C2070, C2075
- Tesla K20c, K20m, K40c
- GeForce GTX580 (3.2GB), GTX680 (4GB), GTX780 (3GB), Titan (6GB)
- Quadro 6000 (6GB)

In theory, BALSA should be able to work with any GPU device supporting CUDA computing capability 2.0 or higher, with 3GB or more on-board graphic memory.

Experiments carried out in this paper were based on GTX680. The number of cores in the GPU device is of essence to the performance of BALSA, but not necessarily *pro rata*.

## RAM

For whole genome analyses of a 50-fold human sample, BALSA requires ~46GB of RAM at peak. Extreme test on a 480-fold human genome dataset shows that BALSA costs ~57GB of RAM at peak. A system with 64GB of RAM or more is recommended for running BALSA optimally.

## IO

As a ballpark figure, if there are *X* GBs of gzipped (compression level 5) FASTQ files with 100bp paired-end reads for a human genome, BALSA roughly does the following:

- Reads *X* GBs of FASTQ files
- Reads 7 GB of 2-way BWT indexed reference
- Writes 2*X* GBs of temporary data
- Reads *X*/15 GBs of temporary data
- Writes 20 GBs SNAPSHOT data (optional)

BALSA was designed with good pipelining practice, which sufficiently overlaps the CPU time, GPU time and IO time. Performance difference between using a recent 3T mechanical hard drive (~140MB/s read and write) and two stripe-arrayed solid-state disk (~1TB/s read and write) is unnoticeable. However, we do not suggest using BALSA with diskless workstations, where temporary data have to be written to network attached storage, as it’s inefficient and the excessive random accesses by modules such as realignment will add large pressure to the storage.

## Tweak

### Reducing Linux swappiness

BALSA is designed to take the full advantage of the hardware resources available on the processing node. On systems with default Linux configuration, this causes the operating system to swap pages out to make room for the IO cache, when the BALSA gets to memory/IO intensive stages. Since BALSA operates on data volumes that normally exceed the amount of RAM of an average system, there is little or no benefit from IO-caching the data BALSA reads and writes.

Reducing the value */proc/sys/vm/swappiness* to 10 or lower from the default 60 solves the problem.

### Using Transparent Huge Pages

Huge pages can improve performance through reduced page faults (a single fault brings in a large chunk of memory at once) and by reducing the cost of virtual to physical address translation (fewer levels of page table to be traversed to get to the physical address). With the large memory footprint and intensive localized memory access natures of BALSA, enabling Transparent Huge Pages feature accelerates BALSA by 20% to 30%. Transparent Huge Pages is a feature enabled by default with 2.6.32 or newer kernels in RHEL or, 2.6.38 or newer kernels in Debian.

## Critical parameters in configuration file

*statMapqThreshold* – Mapping quality score threshold. Reads mapped with mapping quality score lower than the threshold would not be considered for pileup (updating the Counters).

*statTrimSize* – Size of trimming of alignment results. *statTrimSize* at the both edges of a aligned read before soft-clipping would not be considered for pileup (updating the Counters).

*statSoftClipThreshold* – Only Soft Clipping with length ≥ the threshold will be considered (updating the *SoftClipCounters*).

*BalanceSubError* – “Balance Substitute Error” - the error rate of SNP, further defined as *P_s_* in section 8.2.

*UnbalanceSubError* – “Unbalance Substitute Error” - the error rate of Indel, further defined as *P_ID_* in section 8.2.

*RA_MaxBestPattern* – Maximum number of candidate Indel patterns to test in a realignment region, details in section 6.4.

*RA_SupportThreshold* – Minimum number of supporting reads for a candidate Indel pattern to be valid for realignment, details in section 6.8.

*Weight0, Weight1, Weight2, Weight3* – Bin ranges for transforming base qualities to weighted counts, details in section 4.2.

*IndelQualityThreshold* – Averaged base quality threshold for dividing an Indel into high quality or low quality, details in section 4.2.

# Base Quality Score Recalibration

## Workflow and descriptions

For each lane, the base quality score recalibration is to make the quality scores of each read to be more accurate in that the reported quality score is closer to its actual probability of mismatching the reference genome.

For example, a read could contain reported Q25 bases before recalibration, which seems good. However, it may be that these bases actually mismatch the reference at a 1 in 100 rate, so are actually Q20. These higher-than-empirical quality scores provide false confidence in the base calls. Moreover, as is common with sequencing-by-synthesis machine, base mismatches with the reference occur at the end of the reads more frequently than at the beginning.

To derive the empirical base quality score, we count the number of bases with a reported base quality score and how often such bases mismatch the reference base, excluding loci known to vary in the population, according to the dbSNP database.


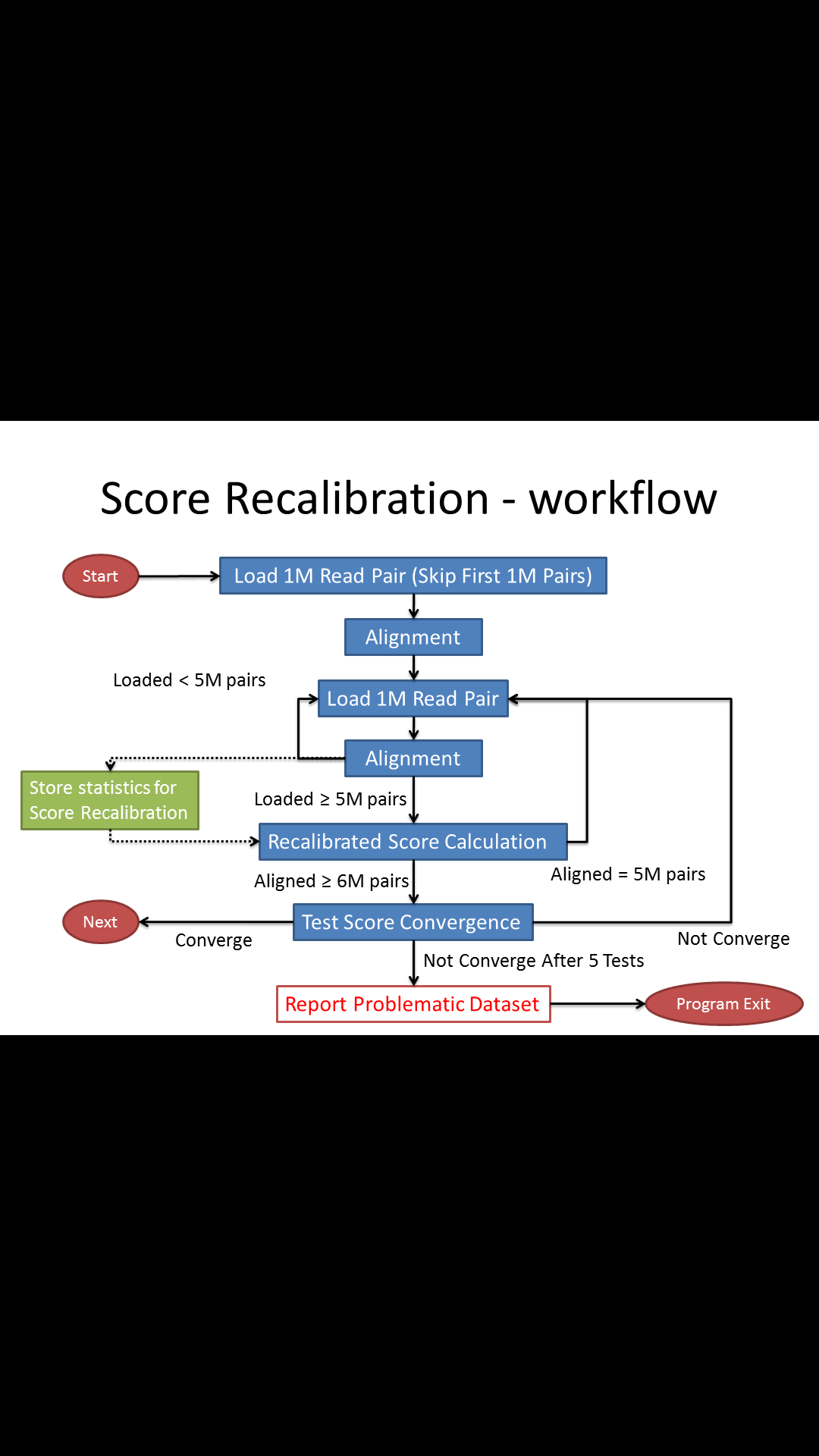


## Reads sampling

For each lane, before formal alignment, reads are sampled for alignment and the alignment results are used for recalibration. To avoid possible bias, the first 1M pairs of reads are skipped and reads are randomly picked afterwards at a rate of 0.25 until 1M pairs of reads are retrieved for alignment. This process continues until the end of score recalibration.

## Score Recalibration and Convergence Test

For each score *i*, i.e. 0 – 40, the number of mismatches and number of nucleotides will be accounted for each aligned reads. Recalibrated score will be calculated by, where and are the number of mismatches and number of nucleotides of base quality score *i* respectively.

Recalibrated scores are calculated after every batch. The first 5M of alignments form the first batch; second batch is the first 6M of alignment (extra 1M to the first batch) and so on. Convergence test is carried out between the recalibrated scores of the current batch and the previous batch. It will proceed to next phase if scores converge in not exceeding 5 convergence tests, otherwise, the dataset will be considered as a problematic set.

Convergence test is defined as followed: let *A* and *B* to be the recalibrated scores from two consecutive batches. *A* and *B* **converge** if for and , where.

## Example pre and post recalibration results

The figure shows the base score recalibration of 8 lanes of 100bp paired-end reads of YH sample sequenced using HiSeq 2000 TruSeq v2. The authenticity of the base qualities improved after recalibration.


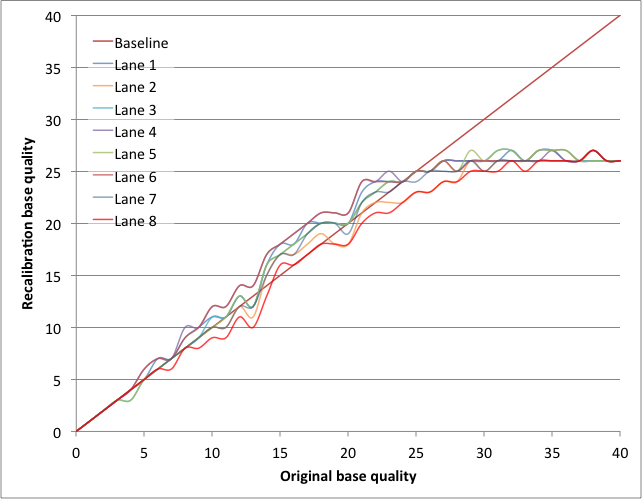


## Future Work

Our current design of the base quality score recalibration module considers only one dimension - the original base quality score. Additional dimensions could be considered including, but not limited to:

1. Sequencing cycles
2. Di-nucleotide context
3. Allele

Empirically, stochastic sequencing errors, where the error rate is coupled with sequencing cycles, could be well recalibrated by just using the original base quality score. However, platform specific systematic bias, such as the increased error rate of continuous G allele reported on Illumina sequencer, can only be well identified by using Di-nucleotide context and Allele. Incorporating these two dimensions into the base quality score recalibration module is our future work. While in the current design, we use at most 12M of reads to reach convergence, the increased dimensions, which in turn requires additional training data to reach convergence, will increase the time consumption dramatically. A gradual converging model that permits less accurate recalibration on the reads loaded anteriorly could be a solution.

# Read Alignment and On-the-fly Pileup

## Workflow and descriptions

Different from the traditional workflows, where aligners output alignments results in files for the subsequent pileup, BALSA maintains a space-efficient data structure in main memory for on-the-fly pileup. The data structure is able to store per-base information including, 1) counts of alleles, 2) counts of strands, 3) reduced base quality and mapping quality, 4) counts of soft-clipping, 5) insertion/deletion types and patterns. The data structure handles vast majority of bases with fixed size memory consumption. The bases with 1) excessive amount of alignments probably due to repetitive sequence and 2) long inserted patterns, require small amount of additional memory to be dynamically allocated at runtime. Albeit fixed size counters are already large enough to handle sequencing depth up to 500-fold, every exceptional base that demands more memory will be fulfilled. Empirically, with a scrupulous design on the data structure, the dynamically allocated memory hardly exceeds 1/10 of the fix size memory consumed even for very deeply sequenced samples. Notably, we do not compromise on the integrity of stored information that may directly or indirectly lead to change of the variant calling results.


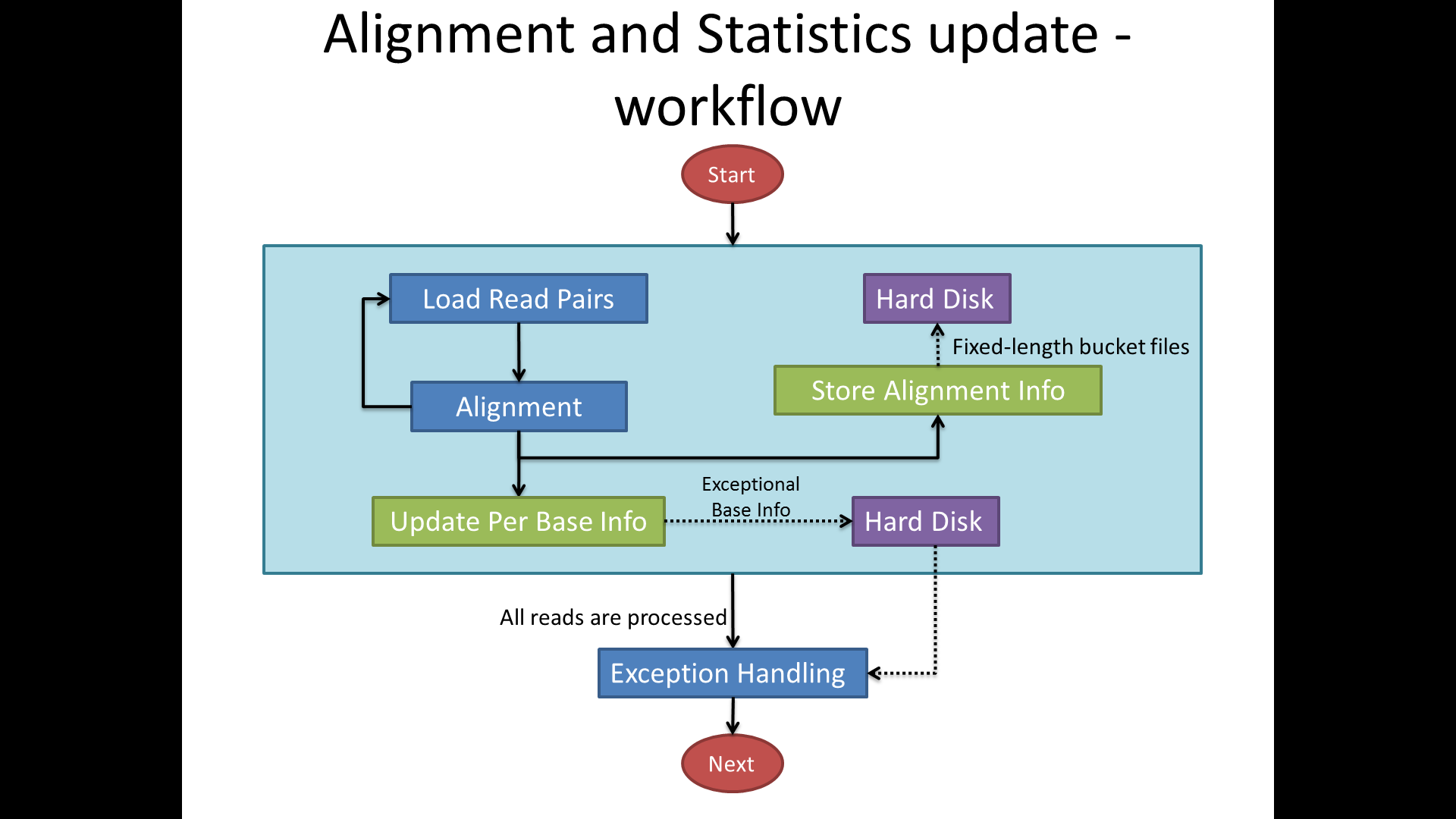


## Per Base Information

### Weighted Count

Each base in an aligned read comprises a recalibrated base quality, ranging from 0 to 40. To store the quality of an aligned base, weights are applied to different ranges of quality scores as follows. The ranges could be modified using parameters.

| Base Quality Scores | Parameter* | Weight |
| --- | --- | --- |
| 0 – 10 | [0 – Weight0] | 0 |
| 11 – 13 | (Weight0 – Weight1] | 1 |
| 14 – 17 | (Weight1 – Weight2] | 2 |
| 18 – 20 | (Weight2 – Weight3] | 3 |
| 21 – 40 | (Weight3 – 40] | 4 |

*See section 2.6 for parameter list

Each aligned base contributes a count to the corresponding allele weighted by its recalibrated base quality. For example, an aligned base ‘A’ with Q25 will add 4 to the “weighted count” counter of allele A, while an aligned base with Q5 will add 0 to the counter.

### Strand Count

According to the alignment result, each aligned base contributes one increment to the positive or negative strand counter of the corresponding allele. Different from weighted count, the increment is always one no matter what recalibrated base quality it has. For example, for a base covered by multiple reads where the recalibrated base qualities are all below 10, the sum of weighted counts could be 0, while the sum of strand counts of both strands exactly reflects the number of reads aligned. Soft-clipping, insertion and deletion are recorded elsewhere and will not contribute count to either weighted counter or strand counter.

### Soft Clip Count

Soft-clipping count will be stored if it exists in an alignment result, i.e. ‘*n*S’ in CIGAR string, while *n* is the length of the clipping and longer than a threshold set by a parameter. Direction of the clipping will also be kept such that the count of head clipping and tail clipping can be distinguished (explained in details in section 4.3).

### Indel Count and Insertion Pattern

Indel information will be stored if it exists in an alignment result, i.e. ‘*m*I’ or ‘*n*D’ in CIGAR string, where *m* and *n* are the length of the insertion and deletion, respectively. Besides the counts of different insertion and deletion types at a base, the patterns of the insertions are also stored in a dynamically allocated data structure (explained in details in section 4.3).sssadfpe at the same position fto 250-fold.ugh rare when fixed size counters are already ucture.onding allele. ng loci known to

For each type, high quality or low quality support, which is determined by the average recalibrated base quality (Q14 by default, set by parameter) of the inserted or deleted pattern, are recorded into two counters respectively.

## Space-efficient Data Structures

Human genome is about 3G base-pairs in size. Amortized *X* bytes consumption for each base will simply lead to 3*X*G memory consumption. To realize on-the-fly pileup, space-efficient data structures were designed with principles including: 1) multiplexing, where a space serves multiple non-overlapping purposes; 2) space utilization is of great essence; 3) minimize overall memory transactions for possible usages; 4) minimize redirections between structures; 5) empirically maintain the ratio of “exceptional bases that require dynamic memory allocation” to “bases that are capable to be handled by fixed size counters” in a range.

Firstly, based on the diploid genome assumption, each genome position ideally have exactly one (vast majority) or two alleles. *Counter*, an 8-byte data structure is designed to store the information including 1) allele type; 2) weighted count; 3) positive strand count and 4) negative strand count of no more than two alleles, each using 4 bytes.


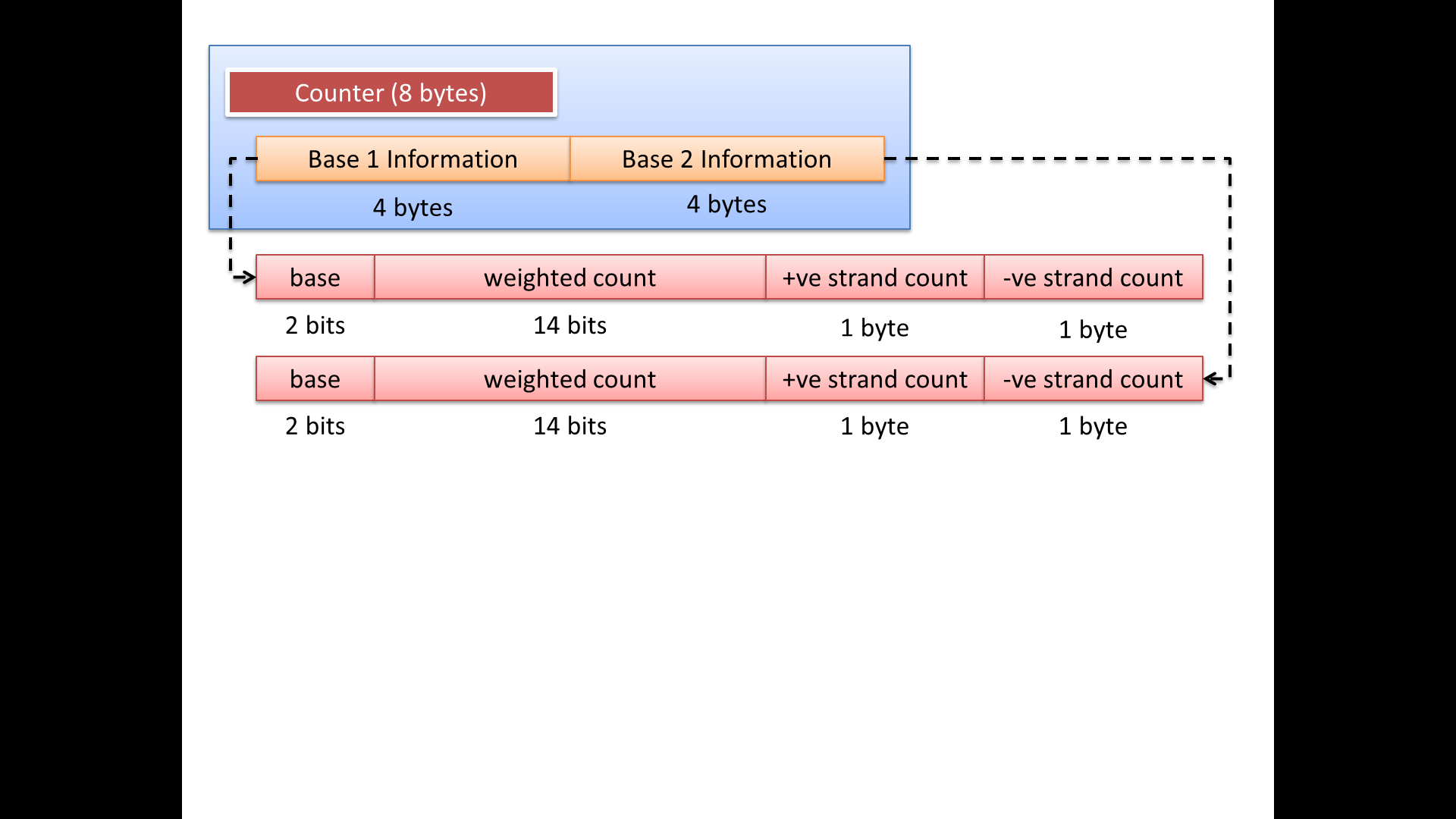


*Base* stores the allele A, C, G or T, represented by “00”, “01”, “10” and “11”, respectively. *Weighted count* stores the weighted sum of allele A, C, G, or T. *Strand count* of both positive and negative strands store the total count of allele A, C, G, or T. The maximum value of each strand counter at this level is bounded to 255.

There are a relatively small number of genome positions (empirically, ~100-200M) that 1) involve 3 to 4 aligned alleles or 2) Indels suggested by alignments. These are regarded as exceptional genome positions. To handle these genome positions, *OverflowCounter*, a 32-byte data structure, is used. Since the existence of these exceptional genome positions are not known in advance, we cannot pre-allocate memory for them; instead, a dynamic array is employed.


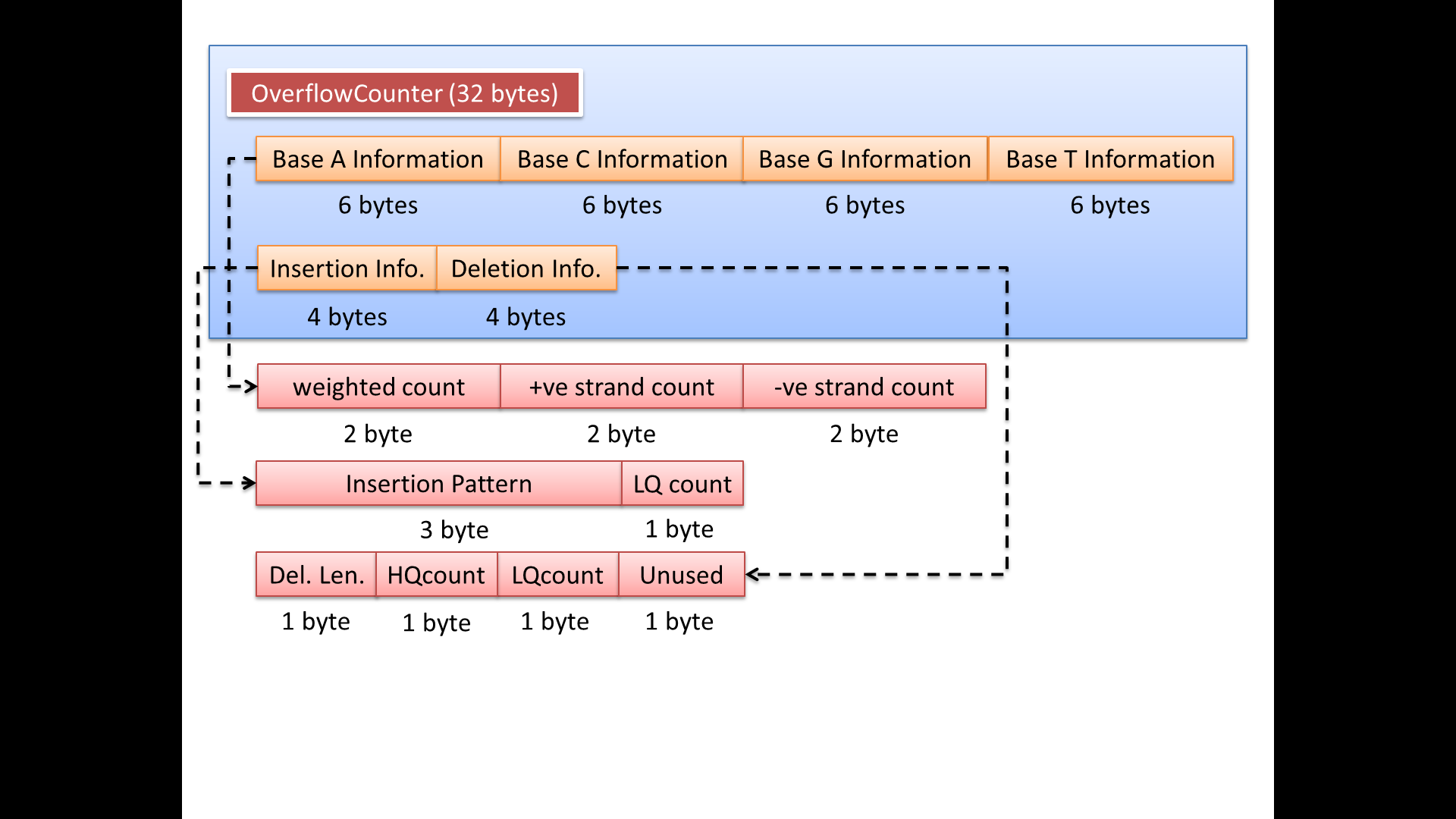


When *OverflowCounter* is used for a genome position, the original 8-byte counter will be transformed to store: 1) the array index pointing to the dynamic array of *OverflowCounter*,2 2) some insertion details including 2) its length and 3) its high quality count.


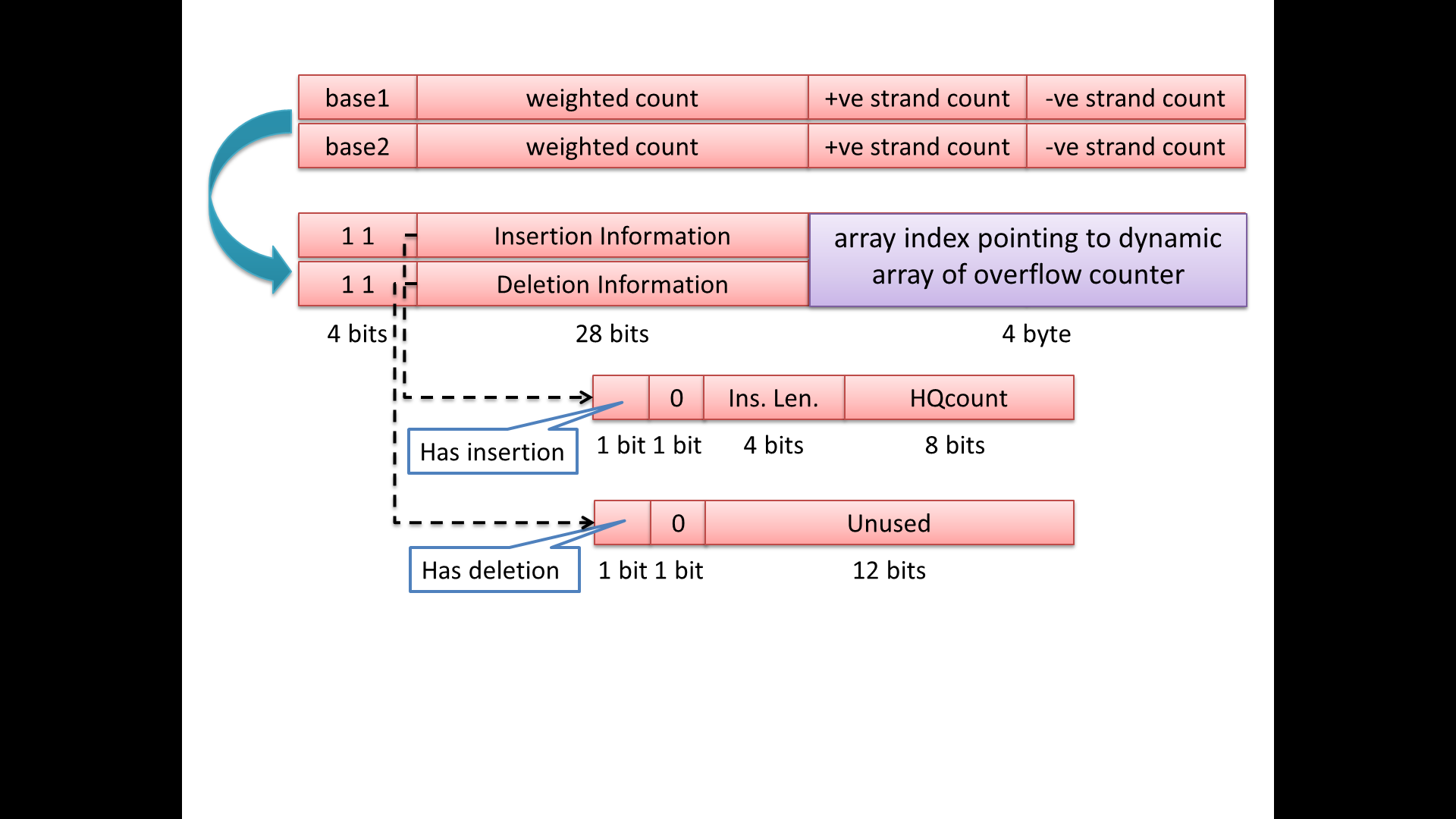


It is not uncommon that 1) more than 1 type of insertions or deletions are suggested by alignments at a genome position, or 2) the inserted pattern is longer than 12 bases (3 bytes), where the *Counter* together with the *OverflowCounter* will be considered insufficient. A “Memory Pool”, where allows arbitrary amount of memory to be allocated for a genome position, is then introduced to handle all remaining circumstances.


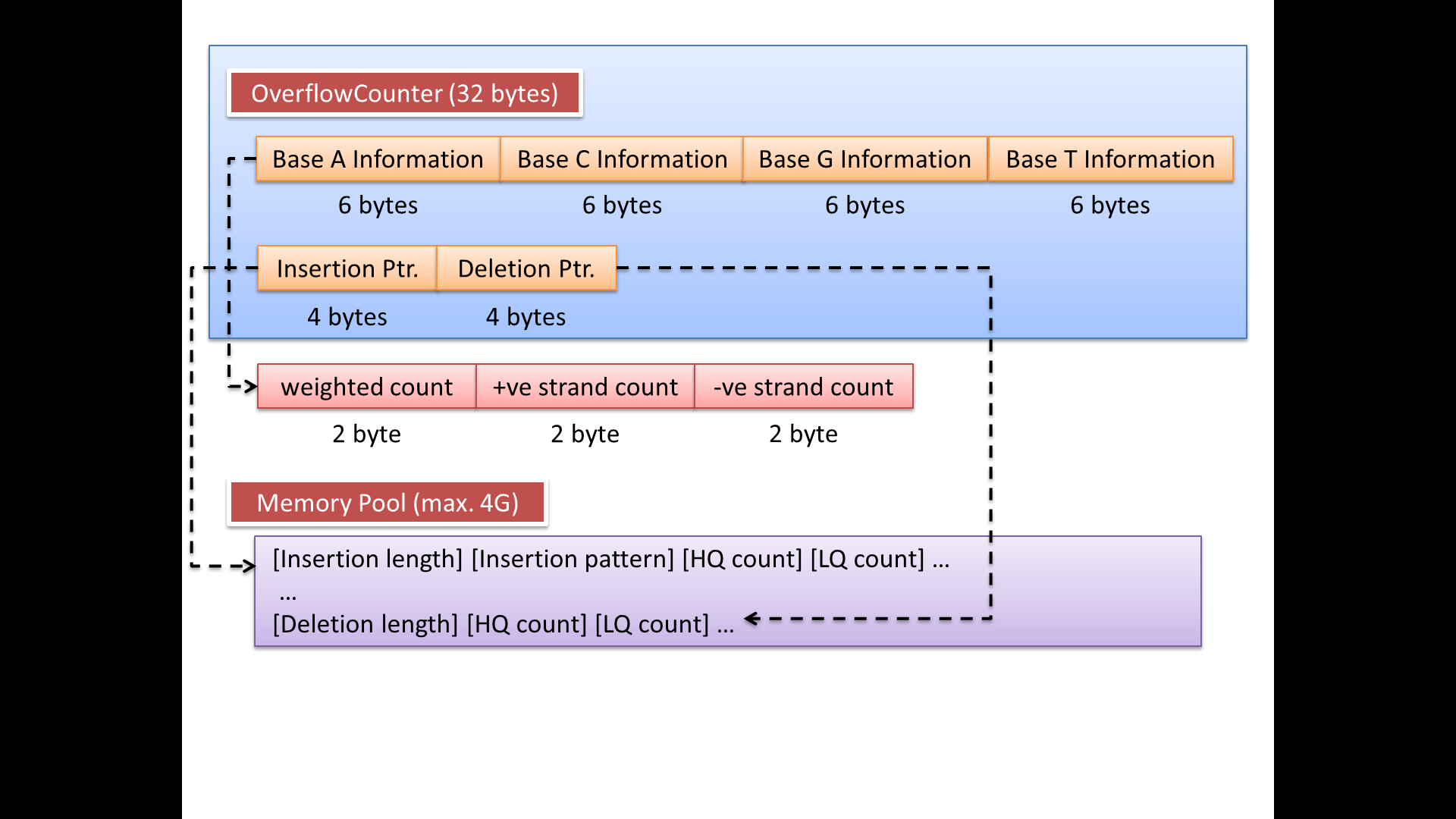


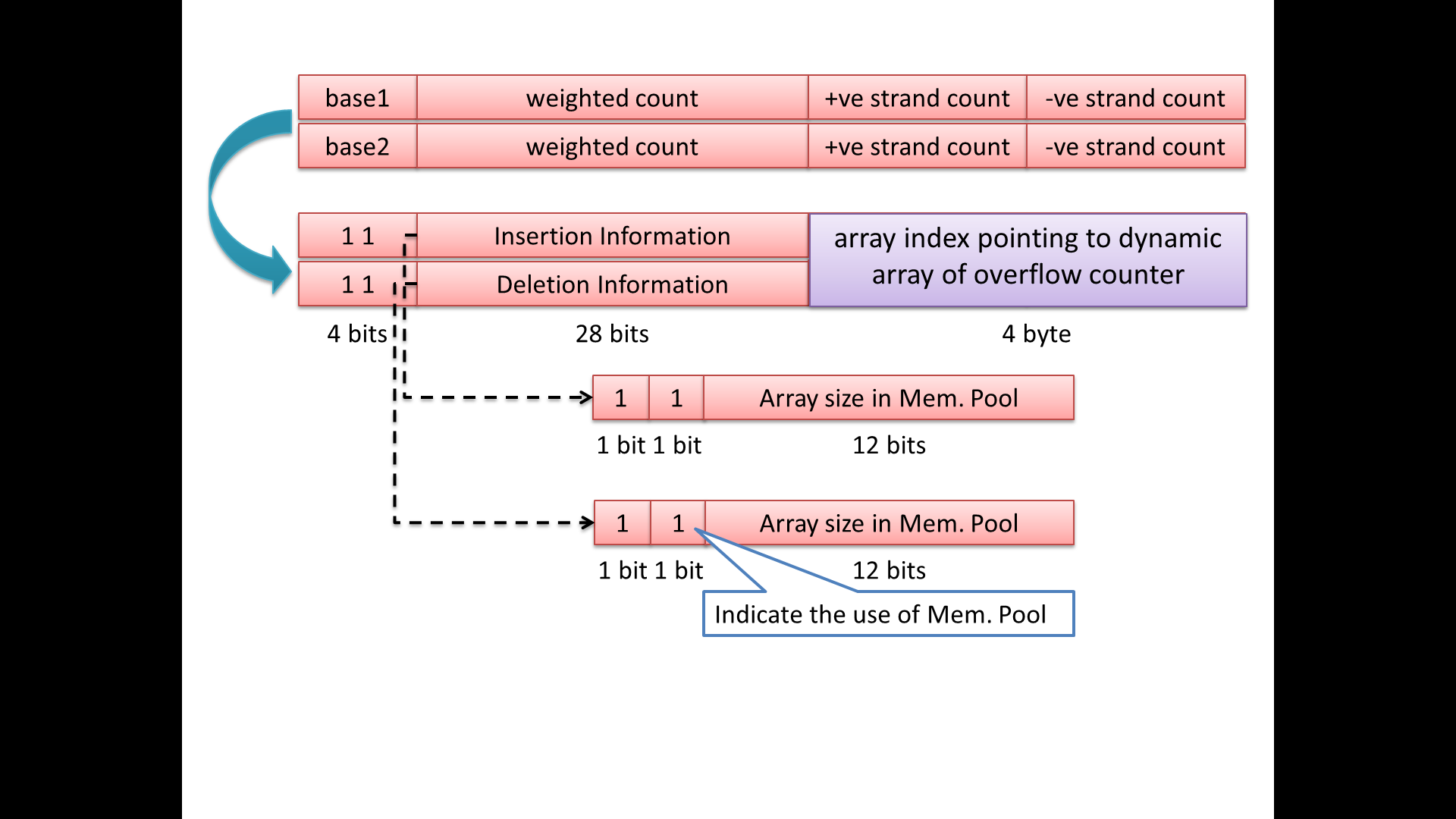


The utilization of a program maintained memory pool enables the use of 32-bit memory pointer, which could be effectively stored by reusing the spaces in *OverflowCounter*. The locality of its data also allows faster memory transaction and on-demand garbage collection. The maximum size of the memory pool is 4G. Although we cannot predict the number of Indels to be stored in the memory pool before running BALSA, and it is not uncommon that, the low quality of the sequencing data or the high heterozygosity of the sample will contribute a high prevalence of Indel signals in alignments. In our experiments, we found that, the utilization of memory pool will hardly exceed 1G even with low quality 150bp short pair-end reads.

Empirically, soft-clipping happens at a small portion of genomic positions, hence it is not necessary to maintain a one-slot-one-position structure to store soft-clipping details. Positions with soft-clipping will be recorded in the format of {position, left count, right count} in a dynamic array, where left and right count refer to the count of soft-clipping at 5’ and 3’-end of the alignments regarding to the positive strand of the reference. The dynamic array is in genome position sorted order.


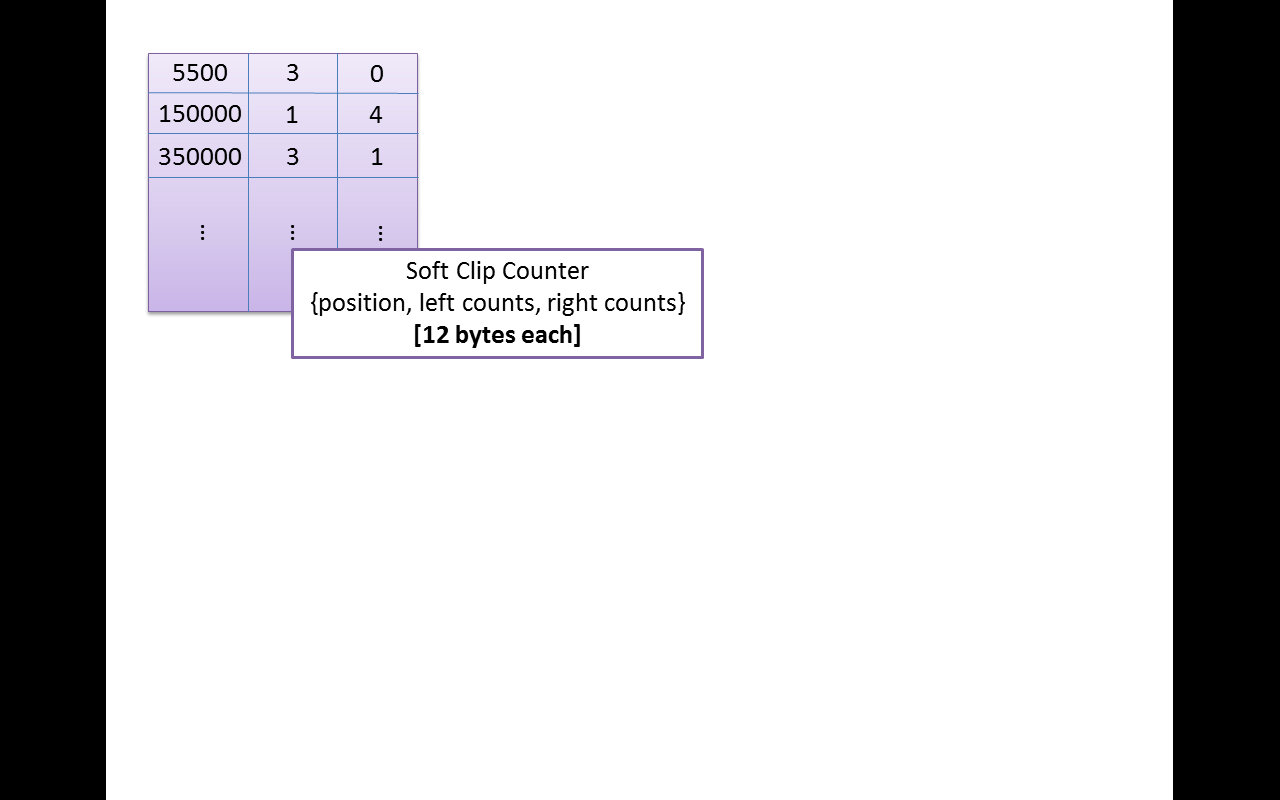


There are two situations that the data structures are unable to hold the information of a genome position, including 1) one or more counts are too large to be held by *OverflowCounter* (>65,535) or *SoftClipCounter*; 2) the reference allele is ‘N’. These genome positions are considered to be invalid thus being marked in a bit vector. BALSA by default excludes these genome positions in variant calling.

## Temporary files

Efficiently storing the intermediate alignment results, which enables fast and random access to the results, is essential for efficiency of the de-duplication and realignment modules introduced later.

For every aligned single-end read or read-pair, we divide the information to be stored into two tiers according to their frequency to be accessed. The alignment information that will be accessed in both de-duplication and realignment modules are “Read ID”, “aligned genome position”, “aligned insert size” (for paired-end mapping only), “mapping quality” and “average base quality of the whole read”, together composing the tier 1 intermediate file. The information that will be access only in realignment module are “read length”, “read bases”, “per base qualities” and “DP alignment details”, together composing the tier 2 intermediate file. The “Read ID” of tier 1 is the index of tier 2. After the alignment, the tier 1 intermediate file will be sorted by “aligned genome position”, hence obtaining a bunch of “Read ID” in a range of genome position can be done by binary searching on the sorted tier 1 intermediate file. For de-duplication, every alignment result in tier 1 will be sequentially visited, while for realignment module, only reads in identified realignment windows (details in Section 0) will be accessed in both tier 1 sequentially and tier 2 randomly.

iningve better queuing ystem to have better querying on the file mediate file will be sorted by "lignment ed realignment w

w
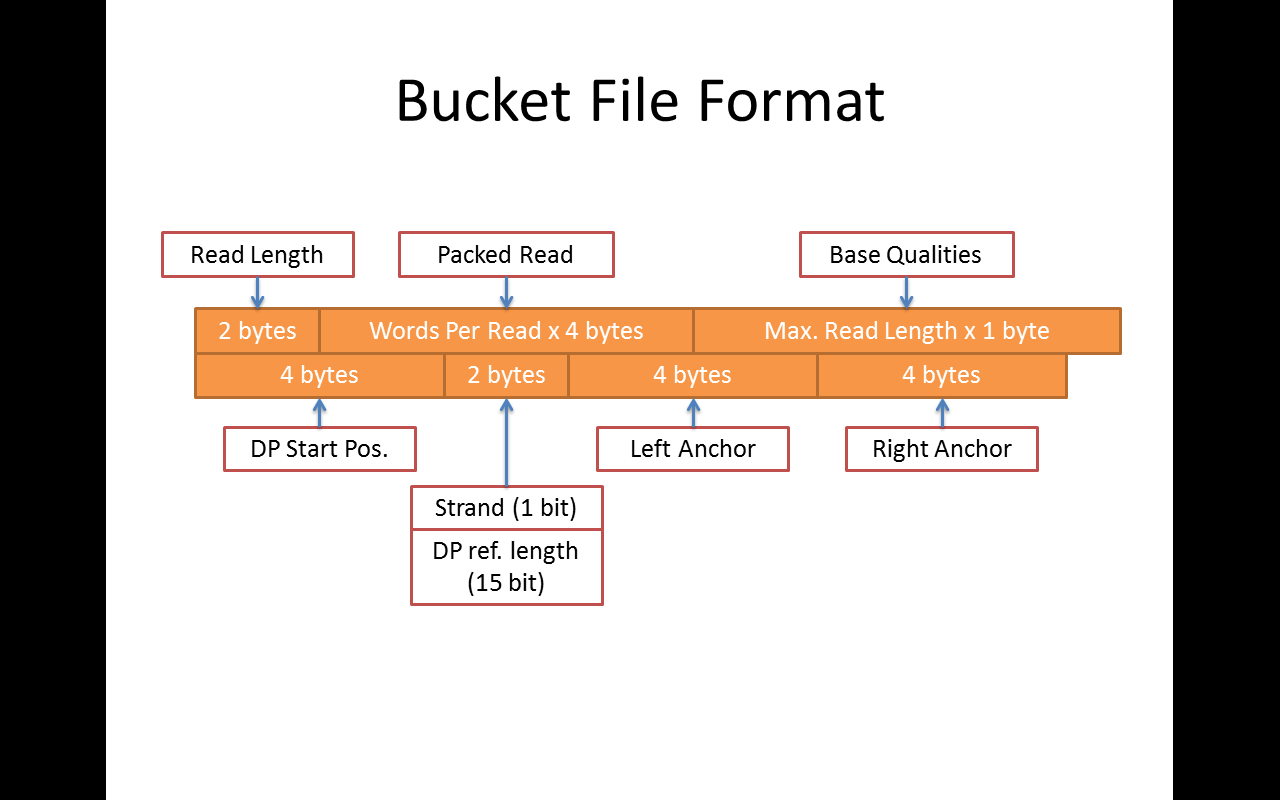


The nature of accessing the data in tier 2 is highly random. Thus by default, we create 512 files (buckets) for tier 2. Read alignments are distributed to one of these 512 files. This enables the operating system to have better queuing on accessing the files.

# De-duplication

## Workflow and descriptions

Duplicately sequenced molecules shouldn’t be counted as additional evidence for or against a putative variant. Reads identified to be duplicates should be removed in advance to realignment and variant calling. In BALSA, de-duplication operates on per lane level at the current stage, and will be extend to be able to operate on per library level in the future.


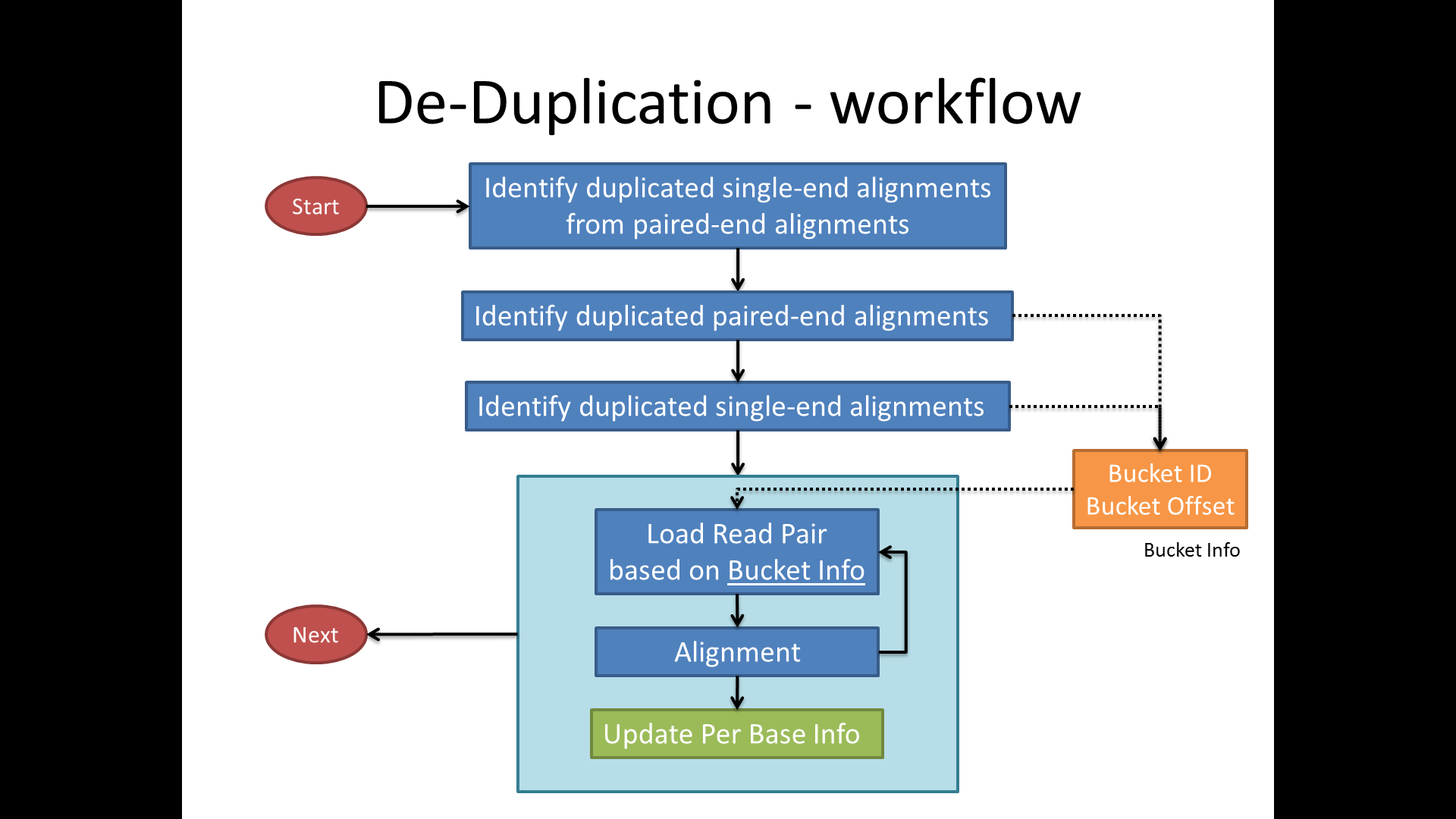


## Duplicate identification


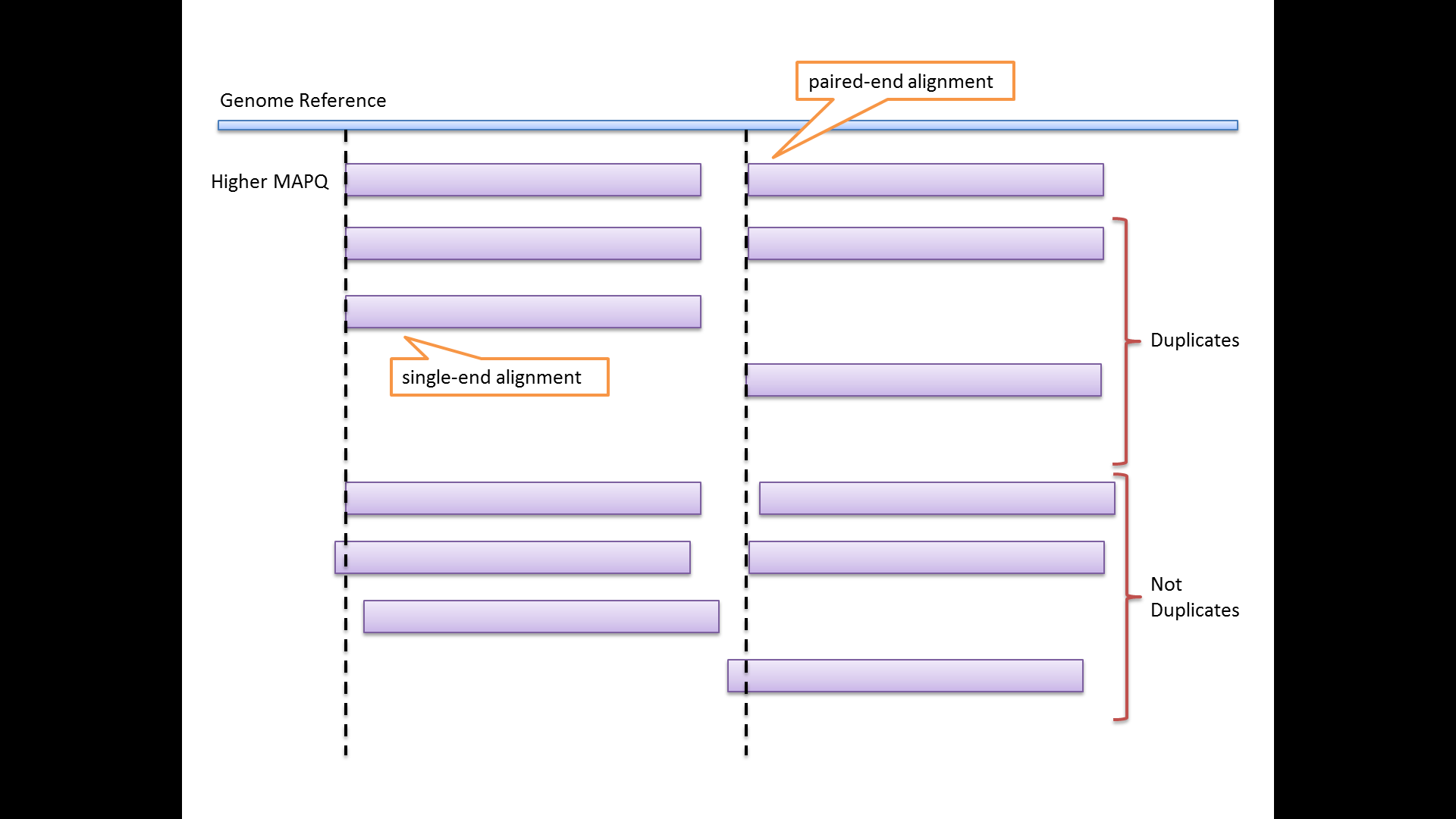


Each paired-end read alignment has two starting positions (each single-end alignment has one starting position). For reads aligned as single-end (not properly paired), 1) if a single-end alignment has the same starting point to another paired-end alignment (any of two), the former will be considered as a duplicate for removal; 2) if a single-end alignment has the same starting point to another single-end alignment, the one with lower mapping quality score will be considered as a duplicate for removal.

For reads aligned as pair-end (properly paired), if two paired-end alignments have exactly the same starting positions, the pair with lower mapping quality score will be considered as a duplicate for removal. Denote that a paired-end alignment has two mapping quality scores; the lower one will be used for comparison.

Counters will be updated with the counts previously contributed by the duplicates removed. Since the CIGAR strings for the alignments are not stored in the temporary files, alignment of the duplicates is performed with banded DP starting from the aligned genome position to regenerate CIGAR strings for Counters update.

## Future Work

There are two types of duplications: 1) molecular duplicates due to PCR artifact, which is the majority and 2) specifically for Illumina’s SBS sequencing, optical duplicates due to clusters very close to each other on a flowcell. The optical duplicates could be detected by identify identical reads with small distant to each other on a flowcell.

# Indel Realignment

## Rationale

The local realignment tool is designed to locally examine and realign reads such that 1) the number of mismatching bases is minimized and 2) the number of Indel patterns and combinations that could be derived is minimized across all reads. In general, a large percentage of regions requiring local realignment are due to the presence of one or several insertions or deletions in the sample’s genome with respect to the reference genome. Such alignment artifacts result in excessive bases mismatching the reference within the misalignment, which may eventually be mistaken as variants. Moreover, since the read mapping algorithms operate on each read independently, it is impossible to place reads on the reference genome locally optimized such that they fulfill the two requirements mentioned during alignment. Consequently, even when some reads are correctly mapped with Indels, reads covering Indels within two edges of the read are often incorrectly mapped with respect to the true Indels, requiring realignment. Local realignment serves to transform regions with misalignments due to the existence of one or more *de facto* Indels into clean read alignments that reveals these Indels.

## Workflow


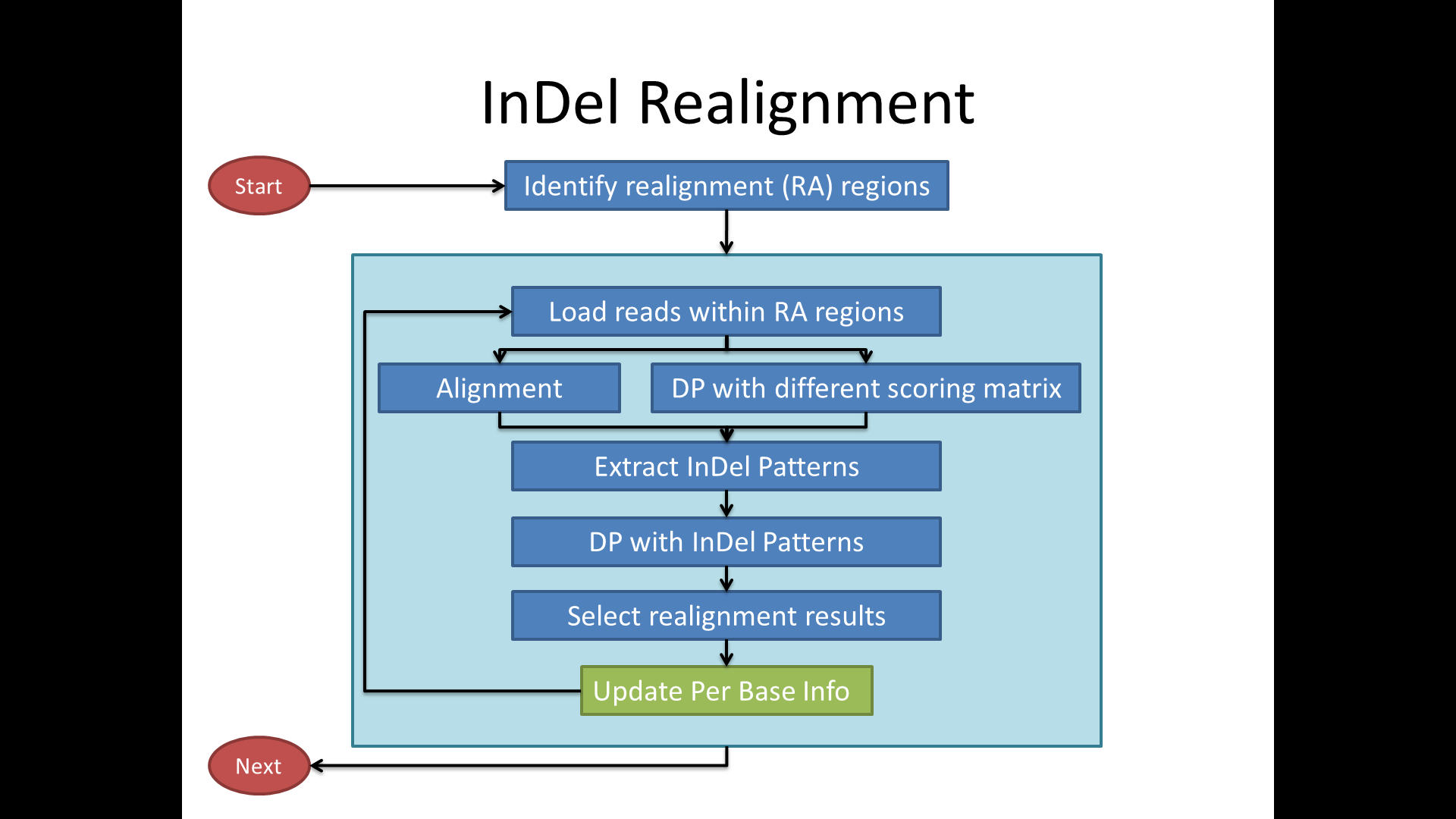


## Realignment Windows and Regions

To determine the reads that need to be realigned, we identify *realignment regions* on the genome where the aligned reads are not piled up with variant signals in a sufficiently consistent way. Roughly speaking, a realignment region is composed of a number of consecutive fixed-size *realignment windows*, in which some bases show alignment inconsistency, e.g., 30% of the aligned reads indicate mismatch at a position while the other 70% of the reads indicate an insertion.

### Realignment windows and triggering conditions

Let *W_size_* be the window size, which is half of the read length, rounded up to the nearest even integer; e.g., for 150 bp reads, *W_size_*=76 bp. Each chromosome of the genome is covered by overlapping windows of size *W_size_*, where the first window starts at the *2W_size_* first position of a chromosome, and consecutive windows are distant apart by a size of (*W_size_*/2) bp. Below we consider a particular window *W*.

Before defining a realignment window, we need some notations, which are derived from the per-base information within *W*. For each position *p* in the window *W*,

- DEL(*p*) is the number of deletions covering position *p*. Here, if a deletion spans from position *i* to *j*, then the deletion covers position *i* to position *j*, and thus contributes 1 increment to DEL(*i*), DEL(*i*+1), …, DEL(*j*).
- INDEL(*p*) is the sum of DEL(*p*) and the number of insertions between position *p*-1 and *p*.
- StrandCount(*p*) is the number of matches/mismatches (i.e., total strand count) at position *p*.
- ReadCount(*p*) is the sum of DEL(*p*) and StrandCount(*p*), which equals to the number of reads aligned over position *p*.

Furthermore, we need the notion of a “*mismatch base*” and an “*indel type*”, and their “*support*”. Consider a position *p*, a *mismatch base* is a base aligned to position *p* that is different with the base on the reference genome, and its *support* is the number of reads having that mismatch base aligned to position *p*. An *indel type* is an *insertion type* (with the same insertion position and the same inserted pattern) or *deletion type* (with the same starting position and the same deletion length), and its support is the number of reads whose alignment has that Indel type.

With the above notion, we can define the following notations for a position *p* in the window *W*:

- MismatchType(*p*) is the number of *mismatch bases* at a position *p* whose support is at least 2 and also at least 25% of the StrandCount(*p*).
- IndelType_≥2_(*p*) is the number of insertion types between position *p*-1 and *p* and the *deletion types* covering position p whose support is at least 2.
- IndelTypeAt(p) is the number of indel types (i.e., insertions between position *p* and *p*-1 and deletions starting at position *p*) whose support is at least 2, if StrandCount(*p*) is at least 1; otherwise, IndelTypeAt(*p*) = 0.

Furthermore, we can define MismatchType(*W*) and IndelType(*W*) for a window *W*, as follows:

and

We also take the soft-clipping information into account. A soft-clipping signal is a soft-clipping in the alignment of a read, and the position of this signal is the position *p* if the soft-clipping clips the 5’-end a read and the first unclipped base of that read is aligned to position *p*, or the soft-clipping clips the 3’-end of a read and the last unclipped base of that read is aligned to position *p*. Then, we define a count SoftClip(*p*) which counts the number of soft-clipping signals at position *p*.

Based on the above per-base information within *W*, the window *W* is a *realignment window* if it satisfies one of the *realignment conditions* below:

#### Condition 1

(MismatchType(*W*) ≥ 1) AND (IndelType(*W*) ≥ 1) AND (MismatchType(*W*) ≥ 2 * IndelType(*W*)).

Intuitively, multiple *mismatch bases* and multiple *Indel types* supported by aligned read in a window, and *mismatch bases* are two times the *Indel types*.

#### Condition 2

There exists a position *p* in *W* where IndelType_≥2_(*p*) ≥ 2.

Intuitively, two or more *Indel types* supported by two or more reads exist at a position in a window.

#### Condition 3

(IndelType(*W*) ≥ 2) AND (There exists a position *p* in *W* where INDEL(*p*) ≥ 25% of ReadCount(*p*) AND StrandCount (*p*) ≥ 1).

Intuitively, two or more *Indel types* exist in a window and at least a position *p* satisfies 1) ≥25% of aligned reads support an *Indel type* and 2) at least a read aligned with a match or mismatch.

#### Condition 4

Let MaxDelLen(*p*) be the maximum deletion length among all deletion types starting at position *p*. For any integer *k* ≥ 0, let

Then, the condition is that: there exists a position *p* in W where,

INDEL(*p*) ≥ 1 AND StrandCount(*p*) ≥ 1 AND [ (SUM(*p*, 0) ≥ 15% of ReadCount(*p*)) OR (SUM(*p*, 1) ≥ 30% of ReadCount(*p*)) OR (SUM(*p*, 2) ≥ 45% of ReadCount(*p*)) OR (SUM(*p*, 3) ≥ 55% of ReadCount(*p*)) OR (SUM(*p*, 4) ≥ 75% of ReadCount(*p*)) ].

This condition is based on the observation that a long Indel exist at the edges of a read will very likely lead to an alignment of the read with soft-clipping at or near the boundary of the indel. Intuitively, the condition considers the excessive amount of soft-clipping signals flanking a putative Indel signal at a position *p* in a window.

#### Condition 5

There exists a position *p* in *W* such that there is an *insertion type* between position *p*-1 and *p* OR *deletion type* starting at position *p*, which exists in IndelDB and whose support is at least Support(*x*), where *x* is the length of that indel, and Support(*i*) = 5 – *i* for *i* = 1, 2, 3, 4, and Support(*j*) = 1 for *j* ≥ 5.

Intuitively, this condition is analogous to GATK IndelRealigner’s ‘KNOWNS_ONLY’ consensus determination model. If sufficient amount of the alignments in a window could be realigned to support a known Indel from IndelDB, the Indel is very likely to be correct.

#### Condition 6

This condition considers the long known Indels from IndelDB only. The condition is: there exists a position *p* in *W* such that IndelDB contains an Indel type with length greater than 20.

Although BALSA was designed to be skillful in detecting long Indels, the sensitivity may still decrease with the increasing length of an Indel. Intuitively, if IndelDB contains a long indel within a window, no matter if there exists any read supporting the Indel or not, the Indel would be considered as an *Indel type* for realignment.

#### Condition 7

This condition considers only the soft-clipping signals. The condition is: there exists a position *p* in *W* with at least 2 soft-clipping, which are at least 25bp in length.

Long soft-clipping is rare, thus, intuitively, we consider two or more long soft-clippings at the same position in a window as a putative signal of Indel. This condition is by default switched off and works in conjunction with modified DP scoring matrix introduced later in section 6.5.

#### Condition 8

This condition considers all the known Indels from IndelDB. The condition is: there exists a position *p* in *W* such that IndelDB contains a known Indel at *p*. This condition is by default switched off. Notices that this is a very wild condition that schedules realignments to all Indel provided by IndelDB. In real analyses, providing sufficient depth, it is not uncommon ones failing to have even 1 correct alignment indicating an authentic Indel signal in a genome region of following types: 1) polymer run, 2) low complexity regions such as TATA boxes, 3) interspersed repetitive sequences such as SINE and LINE. However, providing an accurate and abundant IndelDB, these blind Indels may be able to be rescued by a thorough attempt through all known Indels, while IndelDB contains known Indels detected by various methods, including variants detected by longer sequencing reads such as Sanger and PacBio, which is supposed to be more accurate.

### Realignment regions

We construct realignment regions for each chromosome, as follows: Suppose the chromosome contains identified realignment windows *W_1_*, *W_2_*, …, *W_N_*, in ascending order of their starting positions. We look for the first window *W_i_*, which is not yet considered. Then, we set it as the first window of a new realignment region. This realignment region is further extended in the following iterative manner to have a size of at most 10 times of *W_size_*. Suppose *W_i+x_* is the last window merged into the current realignment region.

Step 1: If *W_i+x+y_* is a realignment window for some integer *y* in [1, 2], extend the region up to *W_i+x+y_* inclusively for the smallest such *y*.

Step 2: Otherwise, if there exists a deletion starting within *W_i+x_* and ending within *V_i+x+2_*, where *V* is a window not matching any condition for realignment (the ending position of *W_i+x_*  is right next to the beginning position of *V_i+x+2_*), extend the region up to *V_i+x+2_* inclusively.

Step 3: Otherwise, if *W_i+x+y_* is a realignment window for some integer y in [3, 6], extend the region up to *W_i+x+y_* inclusively for the smallest such *y*.

Intuitively, 1) windows not more than 3 read length (6 windows) away y, will be merged; 2) two windows with a deletion crossing the boundary will be merged.

Once a realignment region has a size greater than 10 times of *W_size_*, this realignment window is most likely in repetitive regions of the genome, thus ignored from realignment by default. Noted that Step 3 guarantees that two consecutive realignment regions are separated by at least the length of one read length.

**Maintaining a list of realignment regions.** We maintain a list of all the realignment regions for all the chromosomes, and we also maintain a condition flag for each realignment region, which indicates the conditions satisfied by any one of the realignment windows within the realignment region. This flag will be used in later steps to determine whether further processing, e.g., dynamic programming with modified scoring function, is needed.

## Indel Patterns Extraction

An *Indel pattern* for a realignment region is a candidate sequence (with inserted and deleted bases with respect to the reference) to be realigned with (see below for precise definition). We form a set of *Indel patterns* for each realignment region from three sources, namely, 1) BALSA alignment, 2) the Indel database IndelDB, and 3) dynamic programming (DP) with modified scoring matrix. We only consider non-duplicated alignments covering a realignment region. Eventually, realignment results covering a realignment region must be restricted to follow any one of these *Indel patterns*, including the original reference as one of the candidate *Indel pattern*.

### Alignment, IndelDB, DP with modified scoring matrix

*Indel patterns* are extracted from three sources – Alignment, IndelDB and DP with modified scoring matrix. Noted that BALSA does not maintain the CIGAR strings of the alignments in temporary files; we need to reproduce the alignments so as to extract the *Indel patterns* for the realignment regions.

**Indel patterns from alignments.** For each read pair, if one of its reads is paired up to a realignment region, we align it to re-obtain the CIGAR strings of its alignment. *Indel pattern* are generated from the CIGAR strings, which contains the possible combinations of insertion, deletion and matching. Then, we store at most *x* Indel patterns with the greatest average indel length, where *x* is a user-specified parameter for the tradeoff of realignment efficiency and accuracy.

**Indel pattern from IndelDB.** Within any realignment region, if IndelDB contains any known long Indel (length of at least 20bp), we form an *Indel pattern* containing only that long indel. If there are *x* such long indels, we store all the corresponding *x* *Indel patterns* and do not restrict the total number of them.

**Indel pattern from DP with modified scoring matrix.** As we will see in section 6.5, for some realignment regions, we will realign the reads paired up with them using a DP with modified scoring matrix favoring Indels over mismatches. By default, only DP alignment results with exactly one long indel (length of at least 20bp) will form an *Indel pattern*. Notably, we are very conservative on using the *Indel patterns* derived from DP with modified scoring matrix base on the observation that it’s a double-edged sword, which is able to reveal the correct Indel pattern when default scoring matrix cannot, but will also spawn lots of spurious patterns due to its mercy on Indels. In addition, for each realignment region, we keep only one *Indel pattern* with the longest indel length.

## DP with modified scoring matrix

Recall the condition 7 in section 6.3.1.7 that triggers a realignment window. Even without a single alignment support, recurrent soft-clipping signals may infer a putative Indel signal. This deviation is caused by the scoring matrix used by the dynamic programming module. By default, BALSA uses 1, -2, -3, -1 and 0 (positive score for credit, negative score for penalty) for match, mismatch, gap-opening, gap-extension and soft-clipping, respectively. The higher gap-opening penalty along with the lower mismatch and soft-clipping penalties promote alignments with mismatches over Indels. The default scoring matrix is empirically verified to achieve a best balance between sensitivity and accuracy in human genome. However, in the realignments triggered by condition 7, with the assumption that the deviation is very likely to be trigged by a long Indel, all related reads were realigned using a new scoring matrix 1, 7, 1, 1 and 0, in which the penalty to mismatch is way larger than Indel. This eventually enables the long Indel to be rescued for variant calling.

We only consider realignment regions, which has a realignment window satisfying condition 7, and which does not have any know long Indel from IndelDB. Then, we align each read aligned to the realignment region using DP with modified scoring matrix. Recall that only DP alignment results with a single long indel (length of at least 20bp) are used to form an *Indel pattern*.

## Modified References with Indel patterns

After the extraction of Indel patterns, original reference is modified according to the patterns and forms a set of modified references. Modified reference is a reference with the deletion removed and insertion added. We expect that, if a modified reference is exactly what the sample is (the deletions and insertions are authentic), the reads would be realigned unambiguously without Indel to the modified reference. However, if a modified reference is derived from inauthentic deletions and insertions from incorrect alignments, the reads won’t agree to the modified reference and would be realigned inconsistently. That is, simply stated, “there are thousands of rumors but only one truth”.

## DP alignment on modified reference

### Selecting reads for realignment.

For a realignment region, we realign reads, which is non-duplicate and whose alignment overlaps the realignment region by at least one base. It is possible that, for a read not overlapping a realignment region, will fall in the region after realignment with deletion on reference, thus we extended the beginning of each realignment region by 1.5 time a read length by default. Note that all extended realignment regions are disjoint, as it is guaranteed in section 6.3 that the gap size between two consecutive realignment regions is at least a read length, otherwise, the two extended realignment regions will be merged. Reads will be retrieved from temporary files and pair up with their corresponding realignment region.

### DP realignment

The DP realignment results of each read onto each of the modified reference are saved and we will select the best realignment in section 6.8.

Due to the utilization of GPU for alignment, the time consumption of the DP module was bounded by the maximum length among all modified references. We observe that most of the DP instances are with the length of the modified reference much smaller than the maximum length. Therefore, we speed up the DP module by dividing the DP instances into at most four groups, each group will have modified references similar in length.
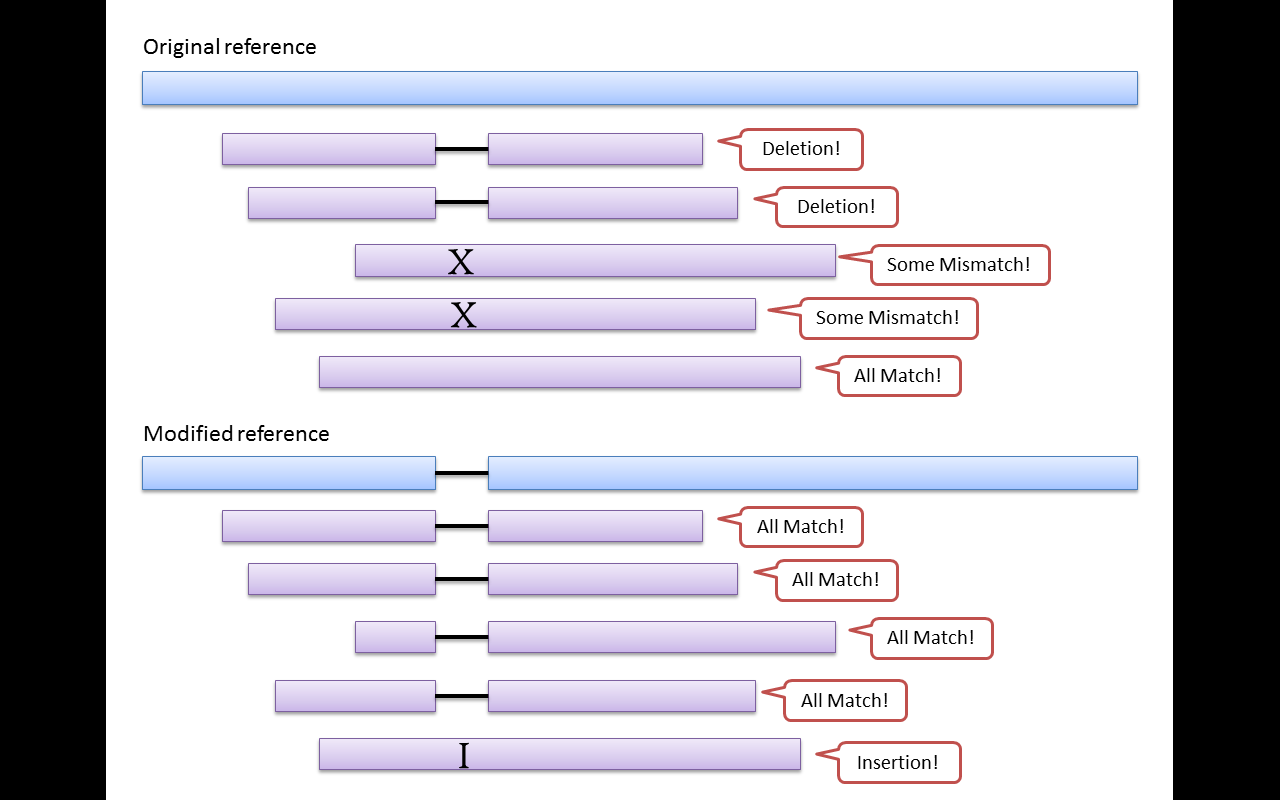


## Best alignment Selection

We need to vote for a best-modified reference by looking at the realignment results in each realignment region. The selection is divided into the two phases as follows:

**Phase 1**: We keep a counter Vote(*m_i_*) for the *i*^th^ modified reference *m_i_* of a realignment region. Let *R* be the realignment results from the modified reference region *m_i_*. For each read associated with the realignment region, suppose the DP alignment result *R_i_* of it over modified reference *m_i_* has the maximum DP score among other results over other modified reference *m_j_*, where *j* ≠ *i*. If this maximum DP score is strictly greater than its original DP score in *R_orig_*, increase Vote(*m_i_*) by 1. A DP alignment will contribute to multiple modified references if they have the same maximum DP scores.


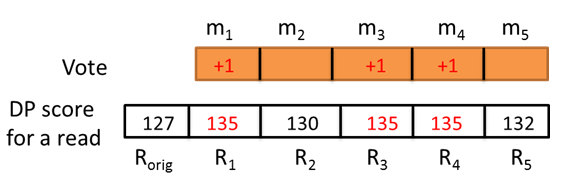


**Phase 2**: Recall that in the array of modified references, each modified reference has a count Count_orig_(*m_i_*), which is the number of the original alignments in that realignment region supporting the modified reference *m_i_*. Let *T* be a user-specified threshold (by default 3). We only consider modified references from *m_i_* where Vote(*m_i_*) - Count_orig_(*m_i_*) ≥ *T*. Such *m_i_* is said to have enough support and can attract enough reads to follow its Indel pattern. For each read associated with the realignment region, we select one best alignment result for *Counter* update as follows:

Consider all valid DP results *R_s_*, where its corresponding *m_i_* is passing the threshold *T*, and its original alignment *R_orig_*. For a DP alignment from *R_s_* with the maximum DP score, if the maximum DP score is strictly greater than its original DP score in *R_orig_*, we select the DP alignment from *R_s_*, otherwise, we keep original DP alignment in *R_orig_* and no update is needed for such read. If two DP alignments in *R_s_* shares a same maximum DP score that higher than *R_orig_*, we select the first one in array context.

# SNAPSHOT

A Snapshot is a dump of all data structures that BALSA needs to work with. A Snapshot after alignment, recalibration and realignment can be considered as a pileup form of all the information from the inputting raw reads. All downstream analyses are designed to be able to start directly from a Snapshot or multiple Snapshots. Specifically, the five data structures to be stored are (for the details of what information each data structure stores, please refer to section 4.3):

- *Counter*
- *OverflowCounter*
- *MemoryPool*
- *SoftClipCounter*
- *Invalid Position bit vector*

Remarks:

*Counter­* – subject to the selection of “Whole Genome Sequencing” or “Exome Sequencing” mode, there are two ways of storing the Counter. For “Whole Genome Sequencing” mode, *Counter* will sequentially store the details of every genome position. For “Exome Sequencing” mode, only [*x*-100, *y*+100], where *x* and *y* are the starts and ends of the specified exome regions, will be stored.

*OverflowCounter* – including *n* dynamic arrays to store the details of positions where *Counter* is insufficient, where n is the number CPU threads used set by user in the configuration (ini) file.

*SoftClipCounter* – Sorted by genome position order.

*Invalid Position bit vector* – If the bit of a genome position is set to 1, the position is considered to be invalid.

# Variants Calling

## Workflow

After score recalibration, alignment, de-duplication and realignment, the consequent SNAPSHOT comprises the aggregated details of base and alignment quality, strand, allele type and count, variant patterns and soft clipping for each site, is used for variant calling.

## 16-Genotype Model

The genotyping model assumes that the sample is a diploid individual, thus the model’s statistical space is the set of un-phased diploid genotypes, G={AA,AC,AG,AT,CC,CG,CT,GG,GT,TT,AX,CX,GX,TX,XX,XY}, where X and Y are the inserted or deleted allele at a site with the highest and second highest depth (i.e. aligned read support), respectively. Different from the traditional 10 genotypes model, which got its prevalence in GATK UnifiedGenotyper, SOAPsnp and other callers, we use a 16-genotypes model, which incorporates both A,C,G,T and Indel alleles. The new model was not only designed to facilitate computation but also dramatically improved the sensitivity and accuracy of variant calling. 5 genotypes {AY,CY,GY,TY,YY}, which implicate an authentic Indel allele with depth lower than the depth of an error Indel allele, were excluded from the diploid combinations of {A,C,G,T,X,Y}. Empirically, these genotypes rarely exist after realignment.

Posterior probabilities of these the 16 genotypes were calculated using a Bayesian model,

…where *F* refers to the observed weighted count of 6 bases at a site in Counter, *P*(*G*), is the prior genotype expectation, *P*(*F*|*G*) expresses the likelihood of the observations and *P*(*G*|*F*) is the posterior probability over genotypes.

There are at least a few reasons you might observe a base call that does not match the reference at a given location: 1) a bad base call during sequencing or primary analysis, 2) a bad alignment resolution of multiple possible alignments, or 3) a genuine variant allele. Because the recalibrated base quality score only help resolve the first cause of error, we specify both error probabilities *P_S_* for ACGT allele (balanced error) and *P_ID_* for Indel allele (unbalanced error). The reason we set two error rates is because of the default scoring matrix applied in the dynamic programming module in alignment and realignment favors balanced error (mismatches) over unbalanced error (Indels). Thus empirically, a *P_S_* should be set higher than *P_ID_*.

If an error occurred (caused by base calling or alignment), assume 1) each of the {A,C,G,T} allele is equal likely to be observed with probability *P_S_* (including the fact alleles), and 2) each of the {X,Y} allele is equal likely to be observed with probability *P_ID_* (including the fact alleles),. We define

…where *m* is the number of {A,C,G,T} alleles in the expected genotype *G*; *n* is the number of {X,Y} alleles in *G*. The default error probabilities used are

When observing a homozygous genotype, we would expect to observe nearly 100% of the homozygous allele. When the allele frequencies of a heterozygous genotype are examined, we expect to observe nearly 50% for each allele. The observed allele frequencies may deviate slightly from the ideal due to call and alignment errors. To test how well an observed depth fits with the expected depth, we used two-tailed Fisher’s Exact Test (FET) with formula defined as below, and take the resulting p-value as the probability of an expected genotype *G*.

Examples of calculating expected genotype probability *P*(*F*|*G*) are given as follows:

The likelihood of a homozygous genotype *G*=AA given observed weighted counts *F* = {*F_A_*, *F_C_*, *F_G_*, *F_T_*, *F_X_*, *F_Y_*} from a site in Counter can be expressed as:

The likelihood of a heterozygous genotype *G*=CG given observed weighted counts *F* = {*F_A_*, *F_C_*, *F_G_*, *F_T_*, *F_X_*, *F_Y_*} can be expressed as:

…where,

*P_hom_* is the probability of observing the allele matching a homozygous genotype,

*P_het_* is the average of the probabilities of observing each of the two alleles of a heterozygous genotype,

*P_e_* is the probability of observing all alleles otherwise exist in the expected genotype *G*,

Given 1) the expected rate *θ* of single nucleotide differences between two unrelated haplotypes, 2) the expected rate *ω* of single Indel differences between two unrelated haplotypes and 3) the expected transition to transversion ratio (Ti/Tv) *ε*, where transition is expected to occur more frequently in real organics, the genotype categories used to describe the model’s prior distributions are as follows:


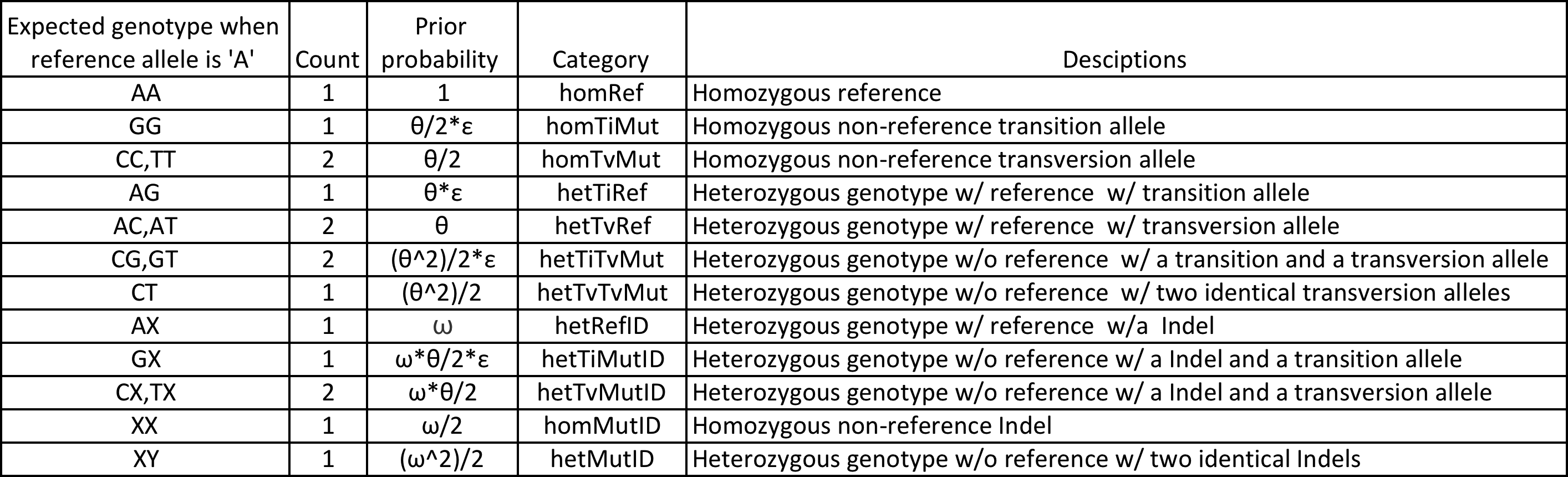


The default expected rates used are

…where *ε* is set for human and subject to change from species to species.

The resulting genotype *G_max_*, is assigned using the expected genotype with maximum posterior probability.

## Variant classification using Random Forest

The purpose of the variant classification is to assign a well-calibrated probability to each variant call in a call set. You can then create highly accurate call sets by filtering based on this single estimate for the accuracy of each call. The approach taken by variant classification is to develop a continuous, covarying estimate of the relationship between variant metrics (such as ReadPosBias, DepthBias, QD, GCPercent, OptSuboptRatio, HRun, etc. Availability of metrics further introduced in the “VCF output” section) and the probability that a variant is a true genetic variant versus a sequencing or secondary analysis artifact. Our model based on random forest is determined adaptively based on 1) “true sites”, typically those sites found or verified to be polymorphic in dbSNP v129, HapMap 3, Omni 2.5M SNP chip array and Mills and 1000G gold standard indels, and 2) “false sites”, dynamically selected from the sites with multiple metrics fall in its worst 5%. This adaptive error model can then be applied to both known and novel variants discovered in the call set of interest to evaluate the probability that each call is real.

### Features used for model training

- Total depth, [0,+∞];
- Depth of the allele with maximum depth, [0,+∞];
- Depth of the allele with second maximum depth, [0,+∞];
- Reference allele {A,C,G,T};
- Strand bias, [-1,1];
- Read position bias, [-1,1];
- Left GC percentage, [0,1];
- Right GC percentage, [0,1];
- Left average base quality, [0,40];
- Right average base quality, [0,40];

### Features used for selecting “false sites”

- Strand bias, [-1,1];
- Read position bias, [-1,1];
- Total depth, [0,+∞];
- Left average base quality, [0,40];
- Right average base quality, [0,40];

### Model training details

SNPs and Indels were identified from YH sample with ~77x 150bp paired-end reads. SNP database dbSNP v137, IndelDB, 1000G and Mills were used for picking “true sites”.

In total, we have 1,813,021 candidate “true sites” and 29,650 candidate “false sites”. All 29,650 “false sites” and 967,936 randomly picked “true sites” were used to form a ~1M training set. A random forest with 96 (set by parameter) trees was built on the training set, giving “out of bag” accuracy at 0.997. The reliability diagram is as follow:


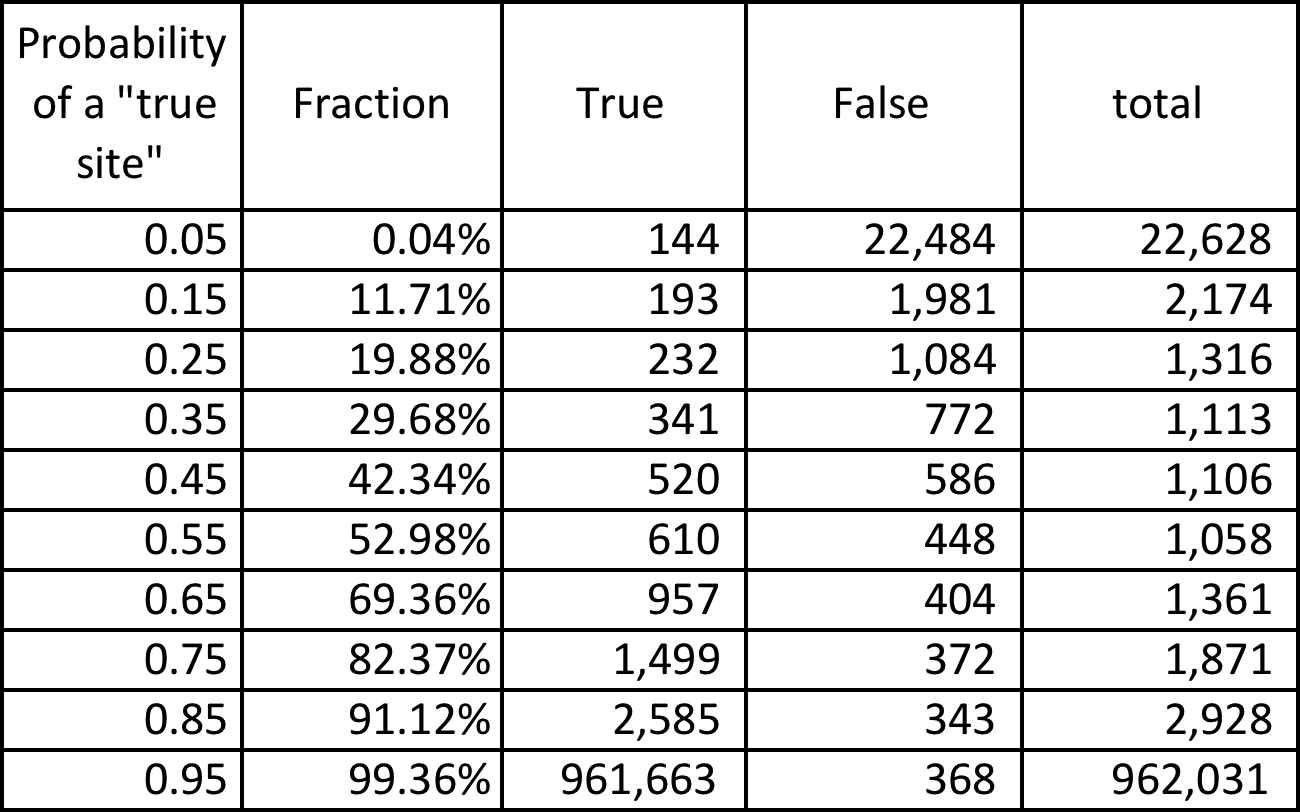


## Output

### Variant Calling Format (VCF)

BALSA outputs a very detailed tab delimited result file for the sites where the resulting genotype *G_max_* is not homozygous reference alleles. The result file can be eventually converted to VCF format version 4.1 using a script. The resulting VCF were tested to be fully compatible with downstream analysis tools such as snpEff, VEP, GATK and online databases including Ingenuity® and GenomeTrax®.

The quality score of each variant was calculated as

…where *P_opt_*(*G*|*F*) is maximum posterior probability and *P_subOpt_*(*G*|*F*) is second maximum posterior probability at a site. The higher the *q*, the lower ambiguity the best genotype has at the site. Limited by computer’s numerical representation, the quality score is ranging from 0 to 3073. The distribution of the quality score of ~5.8M unfiltered mutations (including SNPs and Indels) of YH sample using ~50x 100bp paired-end reads is:

A variety of error cannot be represented in the genotyping models; therefore several metrics are calculated and outputted to the VCF file for users’ reference and could be considered as the training factors for GATK’s VQSR (VariantRecalibrator). The metrics are as follows:


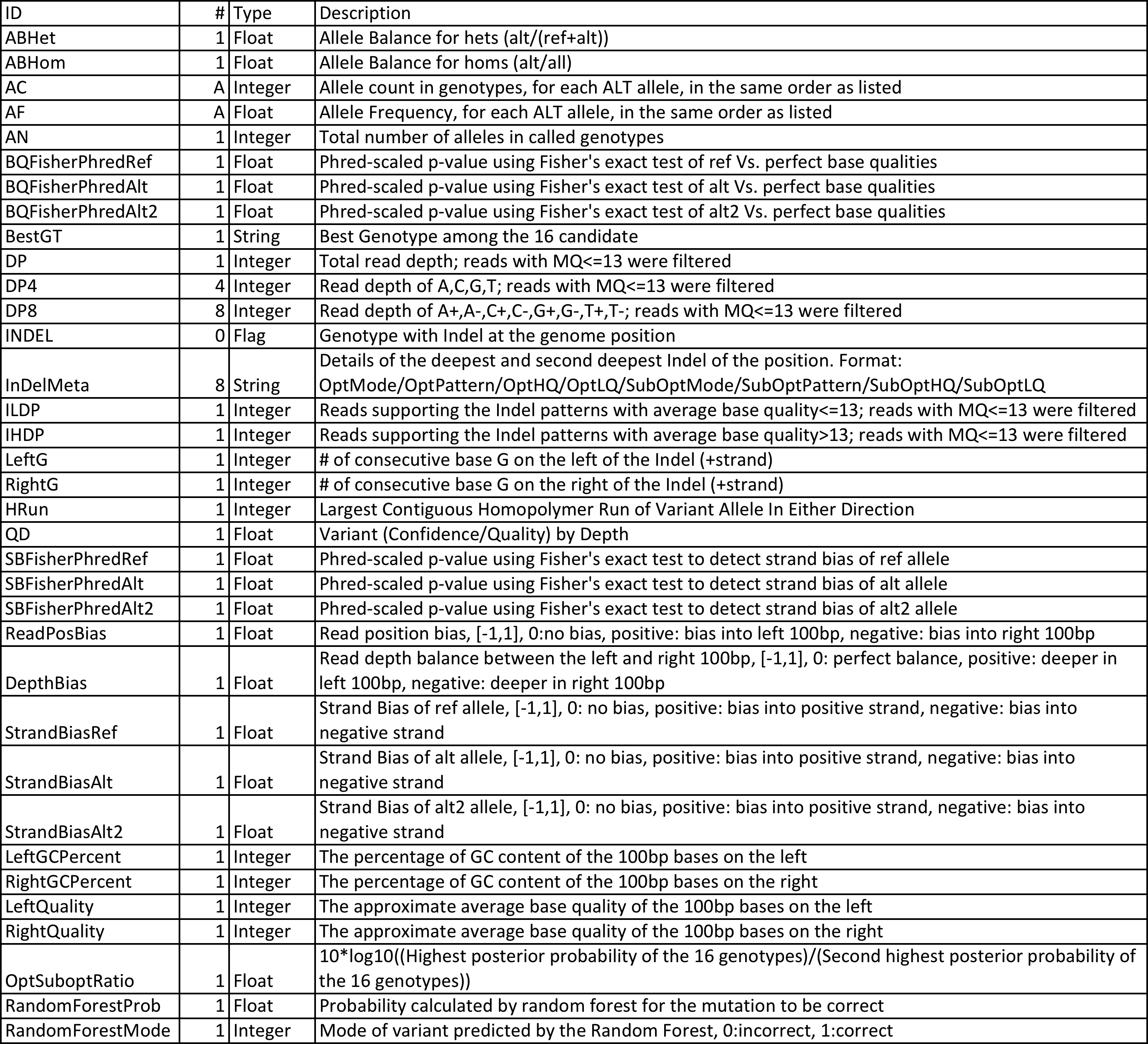


# Somatic Calling

## Workflow

A robust approach to identify somatic mutations is to sequence a paired sample of DNA from normal cells and DNA from tumor tissue from the same patient. The normal tissue can then act as a control against which SNVs detected in the tumor can be screened. Given the two sets of per base information of a tumor-normal paired sample, we focus on the problem of identifying the genotyping difference of the paired sample at every location in the data with coverage.

## Problem Formulation

For a diploid genome we consider all pairs of alleles which gives rise to a set *C*={*AA*,*AB*,*BB*}, where allele *A* indicates that the nucleotide at a position matches the reference genome and *B* indicates that the nucleotide is a mismatch. Now given two diploid samples, the set of possible genotype combinations is equivalent to the Cartesian product of *C* with itself, i.e.

The mutation types of these combinations are (Wildtype: no change; Somatic wildtype normal and variant tumor; Germline: variant normal and tumor; LOH: loss of heterozygosity, heterozygous normal and homozygous tumor; Error: variant normal and wildtype tumor)


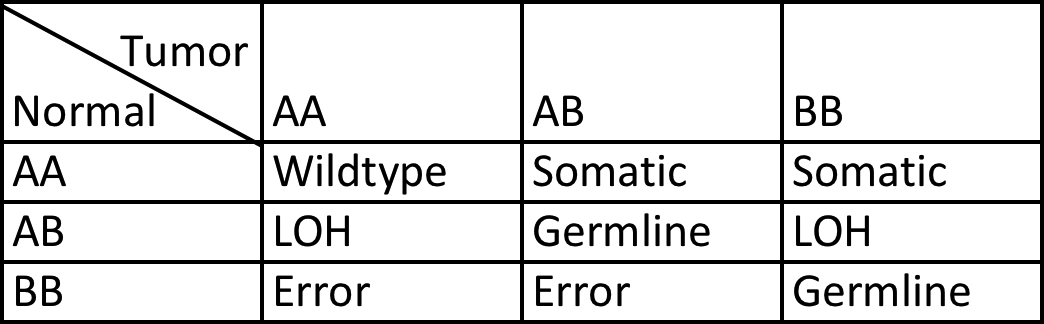


## Algorithm for detecting difference between normal and tumor

To detect somatic mutations, we calculate the probability that a site is not somatic. Give the Counters of both tumor and normal samples, we calculate a somatic score *S* for each site as

…where *L*(*G_i_*) and *L*(*H_i_*) are the posterior genotype probability of the *i*^th^ genotype among the 16 genotypes {AA,AC,AG,AT,CC,CG,CT,GG,GT,TT,AX,CX,GX,TX,XX,XY} from normal and tumor sample, respectively.

*P*(*G_i_*) is the prior probability and calculated as follows:

…where *θ* is the expected rate of heterozygous mutations in the species of interest and *G_R_* is the homozygous reference allele at the site of interest.

*P*(*H*|*G*) is the prior probability of a somatic mutation and calculated as follows:

…where *μ* is the expected somatic mutation rate in tumor.

We only report those sites with *S* equals to or larger than a threshold ß as potential somatic mutations.

The default values used are:

For variation types including Somatic, LOH and Error, in addition, we calculate a p-value *p* to indicate how confident the mutation is using two-tailed Fisher Exact Test (recall the formula shown in the “Variant Calling” section). The details are as follows:

…where *TW_i_* is tumor weighted count and *NW_i_* is normal weighted count of base *i*. *A* denotes reference base and *B* denotes variant base.

For variation types including Wildtype and Germline, *p* is calculated to show how much the variant is different from its corresponding perfect wildtype

…where *TS* and *NS* is the total strand count of tumor and normal respectively. *e* is the error rate *P_err_*, which was previously defined in the “Variant Calling” section.

## Output

BALSA outputs a very detailed tab delimited result file for potential somatic mutations. Output in VCF will be considered in the future.

# CNV

## Workflow and description

BALSA’s CNV detection module is designed to detect somatic CNV, but can also process single genomes.

## Identify candidate segments

We divide the reference genome into segments with user defined size *w* (by default 300bp). Distance of consecutive segments = *w*/2 (by default 150bp). To determine whether a segment is a candidate or not, for each segment, we use Kolmogorov-Smirnov (KS) test to determine whether the depths in the same window of both Normal and Tumor samples differ significantly. If the p-value returned by KS test is larger than *p_cutoff* (by default 0.6), we mark the window as a candidate; otherwise, discard this window.

A *log_2_ratio* (hereafter referred to as *l_2_R*) is derived from each candidate, where:

*log_2_ratio* = log_2_(*RD_T_*/*RD_N_*)

*RD_N_* and *RD_T_* denote the sum of valid read depths in the same window of the normal and tumor sample, respectively.

## Merge overlapping or adjacent candidates

For *k* consecutive candidate segments overlapping each other starting from *i*:, byate segments with overlap:

*k* = 2, if  (*p_merge_* by default 0.27), the *segment_i,i+1_* will be merged;

*k* = 3, if and , the *segment_i,i+1,i+2_* will be merged;

*k* > 3, if , the *segments_i..(i+k-1)_* will be merged.

*normcdf*: Normal cumulative distribution function.

*M*: the mean of *log_2_ratio* in *segments_j_*, where *j* is from *i* to (*i*+*k*-2).

## Adjust segment boundaries

To adjust (refine) merged segment boundary, assume that a merged segment’s left-end is position *i*.

In tumor sample, for all the p-values calculated by KS-test between depths in region [*i-w*/2+*k*, *i*+*k*] and region [*i*+*k*+1, *i*+*w*/2+*k*], where *w* is the segment size (300bp) and *k* is from -*w*/2 to *w*/2, we pick the *k* with minimum p-value, i.e. *i*+*k* is the refined boundary. If equal large minimum p-values exist, we select the first one nearest to the segment boundary.

## Remerge adjacent segments with similar log_2_ratio

This step consists of several rounds of re-merging (by default 2 rounds). The procedure of each round of re-merging is as follows:

Denote:

- Let reg(*i*, *j*) (*i*≤*j*) be the region from the left point of segment *i* to the right point of segment *j*.
- Let l_2_R(*i*, *j*) be the log_2_ratio of reg(*i*,*j*). Hence, l_2_R(*i*,*i*) = l_2_R(*i*) for any *i*.
- Let l_2_R_diff = p_merge * 1.5;

Starting from segment *i*, we consider segment *i*+1, *i*+2, …, one by one. Suppose *k*>*i* is the current segment we consider:

Let *g_k_* be the gap size between segment *k*-1 and segment *k*. Let $\gamma_{k}$ be the ratio of *g_k_* to the average size of segment *k*-1 and segment *k*.

If $\gamma_{k}>1$, segment *k* will NOT be merged to segments*_i_*_..(_*_k_*_-1)_;

Otherwise, if abs(l_2_R(*k*) – l_2_R(*k*-1)) > l2R_diff * (1 - $\gamma_{k}$*)*, segment *k* will NOT be merged to segments*_i_*_..(_*_k_*_-1)_;

Otherwise, if abs(l_2_R(*k*-1,*k*) – l_2_R(*k*-1)) > l_2_R_diff OR abs(l_2_R(*k*-1,*k*) – l_2_R(*k*)) > l_2_R_diff, segment *k* will NOT be merged to segments*_i_*_..(_*_k_*_-1)_;

Otherwise, if abs(l_2_R(*i*, *k*) – l_2_R(*i*)) > l2R_diff, segment k will not be merged to segments*_i_*_..(_*_k_*_-1)_;

Otherwise, segment *k* will be merged to segments*_i_*_..(_*_k_*_-1)_.

## Performance Evaluation

The performance of the CNV detection algorithm was assessed with simulated CNV signals with three different parameters: 1) CNV size, 2) Gap size between two adjacent CNVs, and 3) copy number ratio. Every CNV signal type with a set of parameters has been repeated 100 times and inserted at random genome positions. The false discovery rate (FDR, TP/(TP+FP)) was calculated for every single CNV signals, where a true positive is defined as a detected CNV signal with ≥90% reciprocal overlap with the corresponding simulated signal. The results are plotted as follows. With gap size between 100bp to 175bp, the FDR is high for short CNV signals with subtle copy number changes due to the difficulties of detecting the boundary of these marginal cases. Long CNV signals with copy number loss are with relatively higher FDR due to spurious merge between two adjacent large CNV signals. Notably, CNV signals with gap size longer than 175bp are all being properly detected; this is probably constrained by the offset parameter of two consecutive detection windows. The parameter could be set to lower value for better accuracy but in trade of program running time.


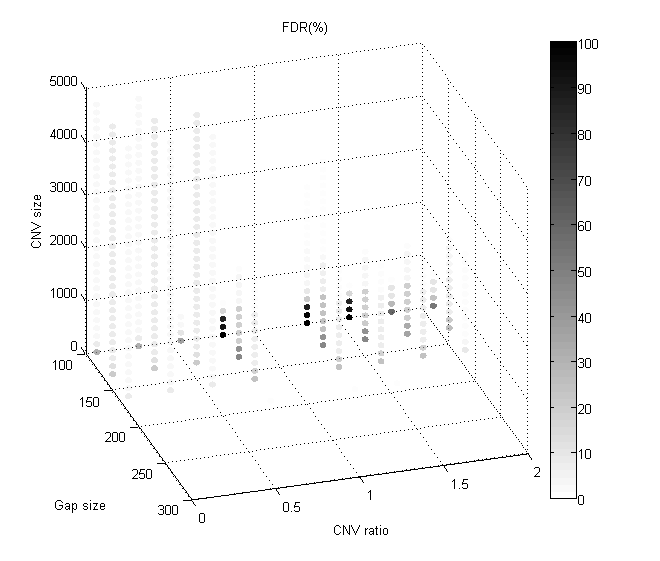


## Output

We output a file with details for the CNVs (merged segments with refined boundaries) we found. Column description as follows:

- *chr*: the chromosome ID of the CNV
- *start*: the starting position of the CNV
- *end*: the ending position of the CNV
- *length*: the length (*end*-*start*) of the CNV
- *avgDepthNormal*: the mean of the depths between *start* and *end* of normal sample
- *avgDepthTumor*: the mean of the depths between *start* and *end* of tumor sample
- *log_2_ratio*: the *log_2_ratio* between *avgDepthTumor* and *avgDepthNormal*
- *log_2_norm*: the base-2 logarithm of (the depths between *start* and *end* of tumor sample)/(overall mean depth of tumor sample)
- p-value: the p-value of KS-test in the between depths between *start* and *end* in tumor and normal samples

Appendix

# Datasets

## YH 100bp and 150bp paired-end read dataset

The 100bp and 150bp paired-end Illumina HiSeq 2000 reads of YH sample were sequenced and deposited to EBI with study accession number ERP001652. The details are as follows:

## Simulated 40x 100bp paired-end read dataset

pIRS v1.1.0 (Hu et al., 2012) was used to simulate reads from the GRCh37 major build human reference genome including 22 autosomes, 2 allosomes and a mitochondrial chromosome. pIRS simulates Illumina reads with empirical base calling and GC%-depth profiles trained from real resequencing data. It claims that the error and base quality distributions as well as coverage bias patterns of simulated reads using pIRS fit the properties of real sequencing data better than existing simulators.

40-fold of 100bp paired-end reads with 500bp mean insert size and 25bp standard deviation were simulated with command:

*pirs simulate -i FASTA1 -I FASTA2 -x 40 -c 1 -o OUTPUT*

…where, *FASTA2* was generated from FASTA1 with SNPs, Indels and structural variations introduced with command:

*pirs diploid -i FASTA1 -a 2.1 -c 2 -o FASTA2*

Eventually, 1) Three lists respectively for the SNPs, Indels and structural variants introduced in *FASTA2* and 2) a pair of gzipped FASTQ file was given.

The Indel list provided by pIRS was left aligned using the following perl script for further comparison. The script takes the Indel list as the first parameter and the reference FASTA’s path (samtools faidx’ed) as the second parameter.

#!/usr/bin/perl -w

use strict;

my $indelFN; my $ref;

($indelFN,$ref) = @ARGV;

die "perl $0 <indel list> <reference>\n" unless defined $ref;

open my $indelFH, "$indelFN" or die $!;

while(<$indelFH>)

{

chomp;

my @F = split;

my $pattern = $F[4];

my $begin = $F[1] - 501; $begin = 1 if $begin < 1;

my $region = "$F[0]:$begin-$F[1]";

my @return = `samtools faidx $ref $region`;

chomp @return;

my $seq = join "", @return[1..(@return-1)];

$seq =~ tr/acgtn/ACGTN/;

my $len = 0;

while($seq=~s/^\w+\K(\w)$//g) {

my $char = $1;

if($pattern =~ /$char$/) {

$pattern = "$char".(substr($pattern, 0, (length($pattern)-1)));

--$F[1];

++$len;

}

else { last; }

}

print STDERR $len if $len > 0;

print join "\t",@F;

print "\n";

}

0;

All simulated datasets are available at <http://bio8.cs.hku.hk/dataset/>BALSA

# Commands

Unless explicitly mentioned, the CPU based aligners and downstream analysis programs were set to use 6 CPU cores (12 threads with Hyper-Threading enabled) without memory limitation on a single computing node with Intel i7-3730k @ 3.2GHz, 64G main memory and 1T two SDD raid0 disk. Programs utilizing GPU were provided with an nVidia GTX680 with 4G on-board memory.

## The BALSA pipeline (version r166)

### Preparing databases for BALSA

Building known SNP database:

*buildSnpDBIndex $Index ${VCF Input} ${Output {prefix}}*

, the command generates two output files, *{prefix}.bv* and *{prefix}.1list*.

Building known Indel database:

*buildIndelDBIndex $Index ${VCF Input} ${Output path}*

Building 1) Exome region database or 2) known gene region database:

*buildRegionIndex $Index ${GFF format Input} ${Output path}*

Currently only support GFF format input.

### From raw reads to variants

BALSA takes in an 8-column multi-row list, which is formatted as following:

*r1.fastq.gz r2.fastq.gz 0 1000 out ID SAMPLE_NAME LB:LIBRARY_NAME\tPL:ILLUMINA\tPU:PLATFORM_UNIT*

where *0* and *1000* represents the minimum and maximum insert size allowed in which an aligned read pair can be classified as “properly paired”.

With the list prepared, we ran BALSA using command:

*balsa pair‑multi genome.fa.index dbSNP IndelDB GeneRegionIntervalList list outputPrefix ‑I ‑L LEN -t tmpPrefix*

where *dbSNP*,*IndelDB*and*GeneRegionIntervalList* are the input files preprocessed dbSNP, known Indel database and the interval list of gene regions, respectively; *outputPrefix* is the file‑name‑prefix of files storing outputs including SNAPSHOT and variant calling results. *LEN* is the maximum read length in the input. *‑I* indicates 64‑base quality encoding. *tmpPrefix*is the file‑name‑prefix of intermediate files, typically on a local hard disk or a SSD.

Converting BALSA output to VCF:

*perl txt2vcf.pl* *BALSAoutput sampleName ref*

where, *BALSAoutput* is the output of BALSA, *sampleName* is the name of sample that would be included into the VCF file, and *ref* is the reference genome fasta file.

### Filtration

The resulting VCF file can be filtered and tagged using following commands (Utilizing GATK’s VQSR and the Random Forest Probability),

*java -Xmx20g -jar $gatk28 -R $ref -T SelectVariants --variant $input.vcf -selectType SNP -o $input.SNP.vcf*

*java -Xmx20g -jar $gatk28 -R $ref -T SelectVariants --variant $input.vcf -selectType INDEL -o $input.INDEL.vcf*

*java -Xmx20g -jar $gatk28 -R $ref -T VariantRecalibrator -input $input.SNP.vcf \*

*-resource:hapmap,known=false,training=true,truth=true,prior=15.0 hapmap_3.3.hg19.vcf \*

*-resource:omni,known=false,training=true,truth=false,prior=12.0 1000G_omni2.5.hg19.vcf \*

*-resource:dbsnp,known=true,training=false,truth=false,prior=8.0 dbsnp_137.hg19.vcf \*

*-an DP \*

*-an ReadPosBias \*

*-an DepthBias \*

*-an StrandBiasAlt \*

*-an LeftGCPercent \*

*-an RightGCPercent \*

*-an ABHet \*

*-an QD \*

*-an OptSuboptRatio \*

*-an LeftQuality \*

*-an HRun \*

*-recalFile $input.SNP.recal -tranchesFile $input.SNP.tranches -mode SNP -nt 12 -minNumBad 5000 -titv 2.15*

*java -Xmx20g $tmp -jar $gatk27 -et NO_ET -K $key -l INFO -R $ref -T VariantRecalibrator -input $input.INDEL.vcf \*

*-resource:mills,VCF,known=true,training=true,truth=true,prior=12.0 Mills_and_1000G_gold_standard.indels.hg19.sites.vcf \*

*-resource:phase1,VCF,known=true,training=true,truth=true,prior=9.0 1000G_phase1.indels.hg19.vcf \*

*-an DP \*

*-an ReadPosBias \*

*-an DepthBias \*

*-an BQFisherPhredAlt \*

*-an SBFisherPhredAlt \*

*-an IHDP \*

*-an ILDP \*

*-an LeftGCPercent \*

*-an RightGCPercent \*

*-an ABHet \*

*-an QD \*

*-an OptSuboptRatio \*

*-an LeftQuality \*

*-an HRun \*

*-recalFile $input.INDEL.recal -tranchesFile $input.INDEL.tranches -mode INDEL -numBad 5000*

*java -Xmx20g $tmp -jar $gatk28 -et NO_ET -K $key -R $ref -T ApplyRecalibration -input $input.SNP.vcf --ts_filter_level 99.0 -recalFile $input.SNP.recal -tranchesFile $input.SNP.tranches -mode SNP -o $input.applyRecalibration.SNP.vcf*

*java -Xmx20g $tmp -jar $gatk28 -et NO_ET -K $key -R $ref -T ApplyRecalibration -input $input.INDEL.vcf --ts_filter_level 99.0 -recalFile $input.INDEL.recal -tranchesFile $input.INDEL.tranches -mode INDEL -o $input.applyRecalibration.INDEL.vcf*

*java -Xmx2g -jar $gatk28 -R $ref -T VariantFiltration --variant $input.applyRecalibration.SNP.vcf -o $output --filterName "bad2" --filterExpression 'RandomForestProb<0.8 || (StrandBiasAlt>0.66 || StrandBiasAlt<-0.66) || (vc.hasAttribute("StrandBiasRef") && (StrandBiasRef>0.66 || StrandBiasRef<-0.66)) || (vc.hasAttribute("StrandBiasAlt2") && (StrandBiasAlt2>0.66 || StrandBiasAlt2<-0.66)) || (DepthBias>0.66 || DepthBias<-0.66) || (ReadPosBias>0.66 || ReadPosBias<-0.66) || QD<30' --filterName "bad1" --filterExpression 'RandomForestProb<0.95 || (StrandBiasAlt>0.5 || StrandBiasAlt<-0.5) || (vc.hasAttribute("StrandBiasRef") && (StrandBiasRef>0.5 || StrandBiasRef<-0.5)) || (vc.hasAttribute("StrandBiasAlt2") && (StrandBiasAlt2>0.5 || StrandBiasAlt2<-0.5)) || (DepthBias>0.5 || DepthBias<-0.5) || (ReadPosBias>0.5 || ReadPosBias<-0.5) || QD<35' --maskName "dbSNP" --mask dbsnp_137.hg19.vcf -cluster 3 -window 10*

*java -Xmx2g -jar $gatk28 -R $ref -T VariantFiltration --variant $input.applyRecalibration.INDEL.vcf -o $output --filterName "bad2" --filterExpression '(((IHDP+1)/(ILDP+1))<2 && ILDP>=3) || RandomForestProb<0.8 || SBFisherPhredAlt>20 || (DepthBias>0.66 || DepthBias<-0.66) || (ReadPosBias>0.66 || ReadPosBias<-0.66) || QD<30' --filterName "bad1" --filterExpression '((IHDP+1)/(ILDP+1))<2|| RandomForestProb<0.95 || SBFisherPhredAlt>10 || (DepthBias>0.5 || DepthBias<-0.5) || (ReadPosBias>0.5 || ReadPosBias<-0.5) || QD<35' --maskName "dbSNP" --mask dbsnp_137.hg19.vcf -cluster 3 -window 10*

To get a clean vcf:

*awk '{if(/^#/){print}else if($7=="dbSNP"||$7=="PASS"){print}else if(($7=="bad1;dbSNP"||$7=="VQSRTrancheSNP99.00to99.90;dbSNP"||$7=="SnpCluster;dbSNP")&&$6>=30){print}' $output > $filteredOutput*

Note that the command retains those known variants marked with “dbSNP” notwithstanding also being marked with “bad1”, “TrancheSNP99.00to99.90” or “SnpCluster”, but requires the variant score larger than 30. A strengthened setting is to retain only variants marked with “dbSNP” or “PASS”.

### Call somatic mutation

Run somatic calling:

*somatic‑caller ${index} ${path_snapshot_normal} ${path_snapshot_tumor} ${somatic_score_threshold} ${output}*

where *somatic_score_threshold* is set to 10 by default.

Divide the Somatic variants into Somatic SNV and Indels:

*awk 'NR==1||($NF=="A"**||$NF=="C"||$NF=="G"||$NF=="T")' $input > $somaticSNV*

*awk 'NR==1||($NF=="X"||$NF=="Y")' $input > $somaticIndel*

Filtration:

Retain those 1) Somatic or LOH variants; 2) 4≤Depth<200; 3) for those variants with somatic score larger than 30, strand bias test with p-value ≥ 0.001, or otherwise strand bias test with p-value ≥0.01.

*awk '($5=="Somatic"||$5=="LOH") && ($17+$18+$19+$20+$21+$22+$23+$24+$27+$28+$31+$32)>=10 && ($17+$18+$19+$20+$21+$22+$23+$24+$27+$28+$31+$32)<200 && ($63+$64+$65+$66+$67+$68+$69+$70+$73+$74+$77+$78)>=10 && ($63+$64+$65+$66+$67+$68+$69+$70+$73+$74+$77+$78)<200 && ((($49>=0.01 && $50>=0.01 && $51>=0.01 && $52>=0.01) && ($95>=0.01 && $96>=0.01 && $97>=0.01 && $98>=0.01)) || ($4>=30 && (($49>=0.001 && $50>=0.001 && $51>=0.001 && $52>=0.001) && ($95>=0.001 && $96>=0.001 && $97>=0.001 && $98>=0.001))))'*

### Call CNV

Run CNV calling:

*cnv-caller ${index} ${path_snapshot_normal} ${path_snapshot_tumor} ${read_length} ${window_size} ${p_cutoff} ${p_merge} ${num_merge_rounds}*

where *read_length* is the longest read length of the input, *window_size* is the size of CNV candidate window, *p_cutoff* is the p-value threshold of choosing CNV window, *p_merge* is the p-value threshold of merging CNV windows, *num_merge_rounds* refers to the number of remerge rounds.

## Running BWA

BWA version 0.7.5a-r405 was used for alignment. Samtools version 0.1.19-44428cd was used for post-processing. The alignment of each lane was executed with the following commands:

### aln

*mkfifo r1.pipe r2.pipe*

*bwa aln ‑I ‑t 6 genome.fasta r1.fastq.gz | bfr ‑b 1G > r1.pipe &*

*bwa aln ‑I ‑t 6 genome.fasta r2.fastq.gz | bfr ‑b 1G > r2.pipe &*

*bwa sampe ‑r '@RG\tPL:ILLUMINA\tID:ID\tSM:SAMPLE_NAME' genome.fasta r1.pipe r2.pipe r1.fastq.gz r2.fastq.gz | bfr ‑b 1G | samtools view ‑S ‑u ‑t genome.fasta.fai ‑ | samtools sort ‑ out.sorted*

*rm r1.pipe r2.pipe*

The alignment of r1 and r2 start simultaneously and pipe the results to post procedures including ‘sampe’, ‘sam2bam’ and ‘sort’ on the fly. The program 'bfr' acts like a buffering dropin replacement for 'cat' (program available at <http://archive.ubuntu.com/ubuntu/pool/universe/b/bfr/bfr_1.6.orig.tar.gz>). It enables the program before a pipe (e.g. aln) to be going on instead of being blocked by the program after a pipe (e.g. sampe). Experiments done by our lab indicated that this is the best way to utilize computational resource as much as possible on a single computing node, which is also the fastest way we can achieve.

### mem

*bwa mem ‑t 12 ‑R '@RG\tPL:ILLUMINA\tID:ID\tSM:SAMPLE_NAME' ‑M genome.fasta r1.fastq.gz r2.fastq.gz | bfr ‑b 1G | samtools view ‑S ‑u ‑t genome.fasta.fai ‑ | samtools sort ‑ out.sorted*

Following completion of the alignment, the output bam is indexed with following command:

*samtools index out.sorted.bam*

## Running SOAP3-dp

SOAP3-dp r176 was used for alignment. SOAP3-dp’s batch-run mode takes in an 8-column multi-row list, which is formatted as following:

*r1.fastq.gz r2.fastq.gz 0 1000 out ID SAMPLE_NAME LB:LIBRARY_NAME\tPL:ILLUMINA\tPU:PLATFORM_UNIT*

…where *0* and *1000* represents the minimum and maximum insert size allowed in which an aligned read pair can be classified as “properly paired”.

With the list prepared, we ran SOAP3-dp using command:

*soap3‑dp pair‑multi genome.fa.index list ‑b 3 ‑I ‑L LEN*

…where *LEN* is the maximum read length in the input.

Following the completion of the alignment, the multiple outputted bam files were 1) sorted individually, 2) eventually merged into a single bam file and 3) being indexed using samtools.

## Running the GATK pipeline and multiple (Ensemble) variant callers

### Post-processing

Following the completion of the alignment, the Picard tool version 1.89 was used to remove duplicate reads.

*java ‑Xmx32g ‑jar MarkDuplicates.jar I=output.sorted.bam O=output.dedup.bam CREATE_INDEX=true*

Indel realignment and base score recalibration is performed using GenomeAnalysisTK-2.8-1-g932cd3a (with database bundle v2.3) with the following commands:

*java ‑Xmx32g GenomeAnalysisTK.jar ‑T RealignerTargetCreator ‑nt 6 ‑R genome.fasta ‑I output.dedup.bam ‑o intervals ‑‑known Mills_and_1000G_gold_standard.indels.hg19.vcf ‑‑known 1000G_phase1.indels.hg19.vcf*

*java ‑Xmx8g ‑jar GenomeAnalysisTK.jar ‑T IndelRealigner ‑model USE_READS ‑known Mills_and_1000G_gold_standard.indels.hg19.vcf ‑known 1000G_phase1.indels.hg19.vcf ‑R genome.fasta ‑‑targetIntervals intervals ‑I output.dedup.bam ‑o output.realigned.bam ‑L chr1*

…where IndelRealigner was ran for each chromosome to facilitate parallelization.

*java ‑Xmx32g ‑jar GenomeAnalysisTK.jar ‑T BaseRecalibrator ‑nct 6 ‑‑knownSites Mills_and_1000G_gold_standard.indels.hg19.vcf ‑‑knownSites 1000G_phase1.indels.hg19.vcf ‑‑knownSites dbsnp_137.hg19.vcf ‑R genome.fasta ‑I output.realigned.bam ‑o grp*

*java ‑Xmx32g ‑jar /nas1/rbluo/soap3‑dp/bin/GenomeAnalysisTK.jar ‑T PrintReads ‑nct 6 ‑BQSR grp ‑R genome.fasta ‑I output.realigned.bam ‑o output.final.bam*

### Ensemble variant caller (Variant calling with 7 callers)

We searched for variants using Atlas2 v1.4.3.r158 (Shen et al., 2010, Challis et al., 2012), GATK HaplotypeCaller and UnifiedGenotyper v2.8-1-g932cd3a (DePristo et al., 2011), Freebayes v0.9.9 (Garrison et al., 2012), Mutect v1.1.4 (Cibulskis et al., 2013), Samtools v0.1.19 (Li et al., 2009) and VarScan v2.3.5 (Koboldt et al., 2012). Unless explicitly mentioned 1) the caller was ran using default parameters, 2) both SNP and Indel results of a caller were used for integration and downstream analyses.

#### Atlas2

Atlas2-SNP and Atlas2-Indel are based on logistic regression model. Illumina mode and a minimum depth of 4 were set for detection. Atlas2 does not provide variant quality and other metric in the output for downstream judgment but uses built-in filters to classify the mutation, printing PASS or FAIL tags in the output.

#### GATK UnifiedGenotyper and HaplotypeCaller

UnifiedGenotyper uses Bayesian likelihood model while HaplotypeCaller combines a local assembler and an affine gap penalty Pair Hidden Markov model to estimate the genotype and its probability. Following completion of the variant calling, variant quality score recalibration is performed. Firstly, SNPs and Indels were split into two separate files; variant recalibration is then performed on the extracted variants with the following commands:

*java ‑Xmx32g ‑jar GenomeAnalysisTK.jar ‑R genome.fasta ‑T VariantRecalibrator ‑input output.final.SNP.vcf ‑resource:hapmap,known=false,training=true,truth=true,prior=15.0 hapmap_3.3.hg19.vcf ‑resource:omni,known=false,training=true,truth=false,prior=12.0 1000G_omni2.5.hg19.vcf ‑resource:dbsnp,known=true,training=false,truth=false,prior=8.0 dbsnp_137.hg19.vcf ‑an QD ‑an HaplotypeScore ‑an MQRankSum ‑an ReadPosRankSum ‑an FS ‑an MQ ‑recalFile SNP.recal ‑tranchesFile SNP.tranches ‑mode SNP*

*java ‑Xmx6g ‑jar GenomeAnalysisTK.jar ‑R genome.fasta ‑T VariantRecalibrator ‑input output.final.INDEL.vcf ‑resource:mills,VCF,known=true,training=true,truth=true,prior=12.0 Mills_and_1000G_gold_standard.indels.hg19.vcf ‑resource:phase1,VCF,known=true,training=true,truth=true,prior=9.0 1000G_phase1.indels.hg19.vcf ‑an QD ‑an FS ‑an ReadPosRankSum ‑an MQ ‑an MQRankSum ‑recalFile INDEL.recal ‑tranchesFile INDEL.tranches ‑mode INDEL*

*java ‑Xmx6g GenomeAnalysisTK.jar ‑R genome.fasta ‑T ApplyRecalibration ‑input output.final.SNP.vcf ‑‑ts_filter_level 99.0 ‑recalFile SNP.recal ‑tranchesFile SNP.tranches ‑mode SNP ‑o output.recalibrated.SNP.vcf*

*java ‑Xmx6g GenomeAnalysisTK.jar ‑R genome.fasta ‑T ApplyRecalibration ‑input output.final.INDEL.vcf ‑‑ts_filter_level 99.0 ‑recalFile INDEL.recal ‑tranchesFile INDEL.tranches ‑mode INDEL ‑o output.recalibrated.INDEL.vcf*

Mutations with “VQSRTranche” larger than 99.0% were removed.

#### Freebayes

Freebayes is a haplotype-based variant detection method based on Bayesian statistical framework. Reads with 1) mismatches more than 10% of the read length; 2) 0 mapping quality; 3) more than 4 separate gaps were filtered. Mutations with ≥4-read support were used for integration and downstream analyses.

#### Mutect

Mutect is a method that applies a Bayesian classifier to detect somatic mutations with very low allele frequency. It was designed to identify somatic variants from paired samples but is able to be configured to detect SNP. The HC filters in Mutect were reported to be able to reduce the false positive rate by an order of magnitude. Mutations failing the filters will be assigned with a “REJECT” tag and be excluded for integration and downstream analyses. Mutect was only able to generate SNP results.

#### SAMtools genotype calling

The ‘mpileup’ command in SAMtools together with the accompanying ‘bcftools’ were used to identify SNPs and Indels at locations with equal or more than 10 reads supporting. The raw results were filtered using “vcfutils.pl varFilter” program with default parameters.

#### VarScan genotype calling

VarScan requires input in pileup format, which was generated with SAMtools’ ‘mpileup’ using parameters 1) minimum mapping quality of one and 2) extended BAQ (Li et al., 2012) sensitivity. We ran VarScan requiring 1) Fisher exact test with a 0.98 significance level and 2) ≥4 reads support. Only Indels identified by VarScan were used for integration and downstream analyses.

### Integrating variant results from 7 callers

#### SNP integration

SNP results of each caller were merged using GATK CombineVariants. SNPs 1) specifically called by only a tool and with variant quality score lower than 50% of all SNPs identified by the tool; 2) with all tools with variant quality score lower than 90% of all SNPs identified by the corresponding tools; 3) with ≤4 reads supporting were filtered. Specifically, as we’ve observed increased artifacts in Atlas2-SNP with high depth, SNPs specifically detected by Altas2-SNP with depth ≥100 but not conforming filtering condition 1, were also filtered.

#### Indel integration

Indel results of each caller were pre-processed by GATK LeftAlignAndTrimVariants and then merged using ‘vcf-merge’ from ‘vcftools’ (<http://vcftools.sourceforge.net>). Indel 1) specifically called by only a tool; 2) with ≤4 reads support, were filtered.

## The Isaac pipeline

Alignment was performed using the Isaac aligner version 13.09.17. The Isaac aligner executable isaac-align was run with following non-default parameters:

*--reference-genome genome.xml*

*--reference-name default*

*--base-calls-format fastq-gz*

*--temp-directory /ssd/*

*--jobs 12*

*--memory-limit 55*

*--base-quality-cutoff 15*

Following completion of the alignment step, variant calling is performance using the Isaac variant caller. The Isaac variant caller workflow creates a makefile for subsequent parallel analysis according to a ini file with following settings:

*isSkipDepthFilters = 1*

*maxInputDepth = 10000*

*depthFilterMultiple = 3.0*

*indelMaxRefRepeat = -1*

*minMapq = 20*

*minGQX = 30*

*isWriteRealignedBam = 0*

*binSize = 25000000*

## Running Mutect for somatic mutations

Following the completion of the alignment and GATK pre-processing of both normal and tumor samples, the somatic mutation calling using Mutect version 1.1.4 is performed with the following command on all chromosomes:

*java ‑Xmx4g ‑jar muTect‑1.1.4.jar ‑‑analysis_type MuTect ‑‑reference_sequence genome.fasta ‑‑cosmic b37_cosmic_v54_120711.vcf ‑‑dbsnp dbsnp_132_b37.leftAligned.vcf ‑‑input_file:normal output.final.normal.bam ‑‑input_file:tumor output.final.tumor.bam ‑‑out chr1.out ‑‑coverage_file chr1.wig ‑‑intervals chr1*
