# Supplementary figures and images for: BALSA: integrated secondary analysis for whole-genome and whole-exome sequencing, accelerated by GPU
Source: PeerJ. 2014 Jun 3;2:e421. doi: 10.7717/peerj.421 (PMC4060040; doi:10.7717/peerj.421)

# Comparing BALSA to Individual callers

SNP

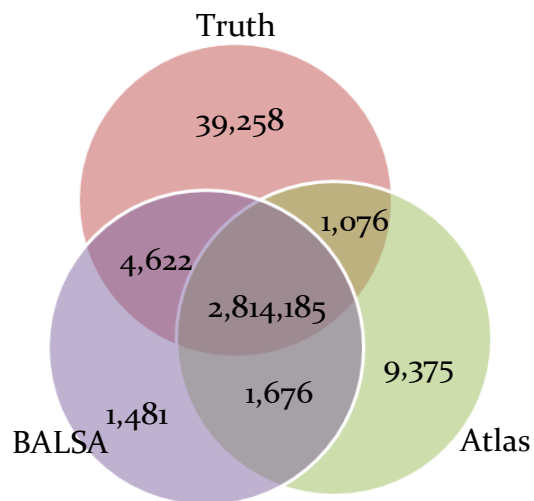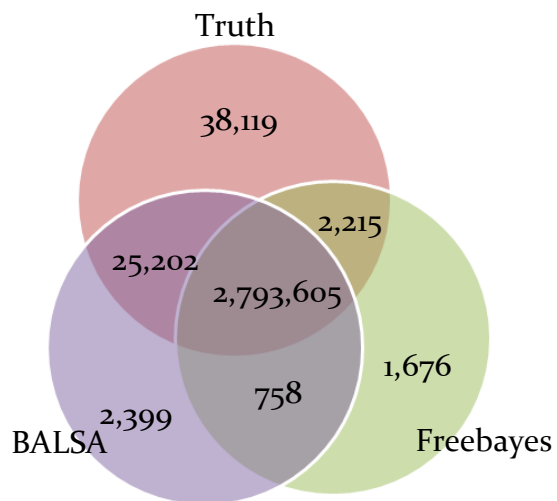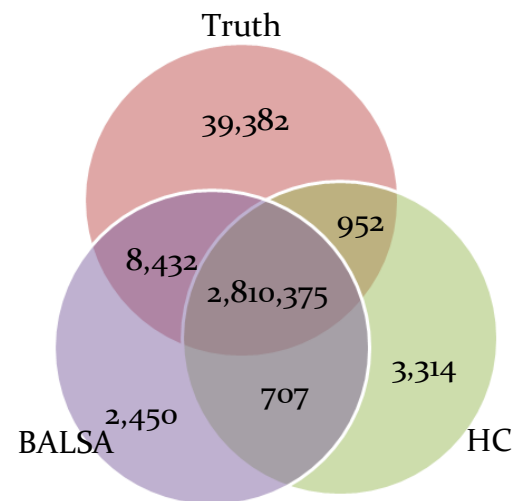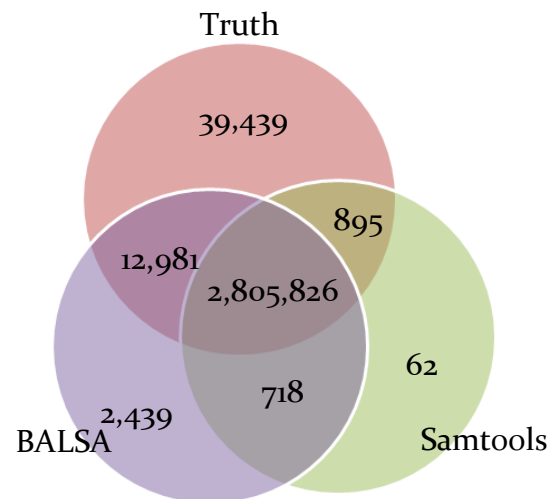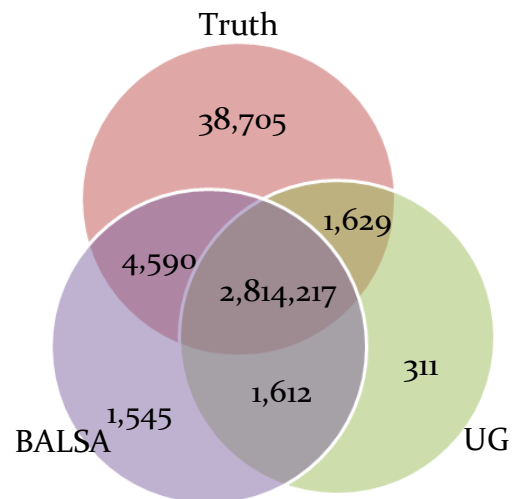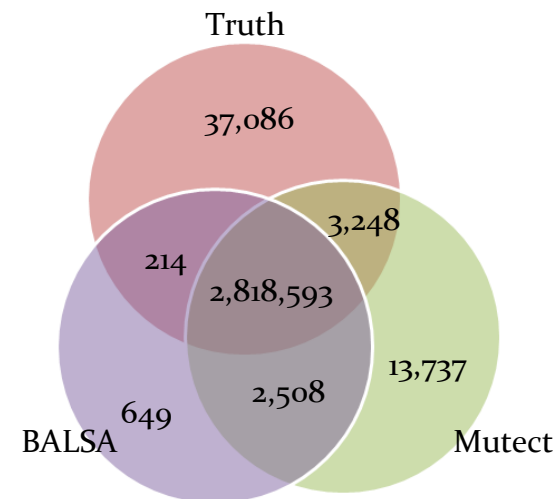

Supplement: Figure S1 [file peerj-02-421-s002.pdf]

# Comparing BALSA to Individual callers

Indel

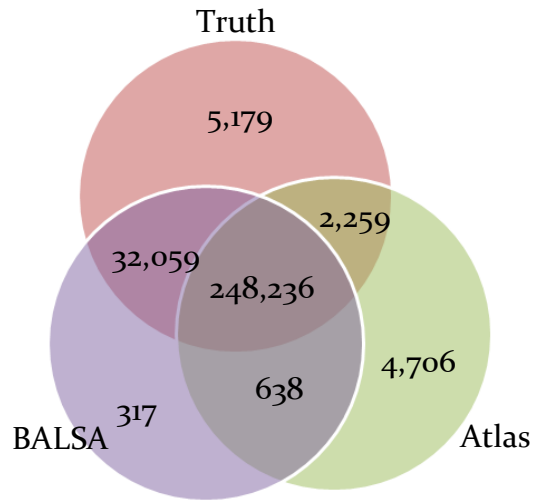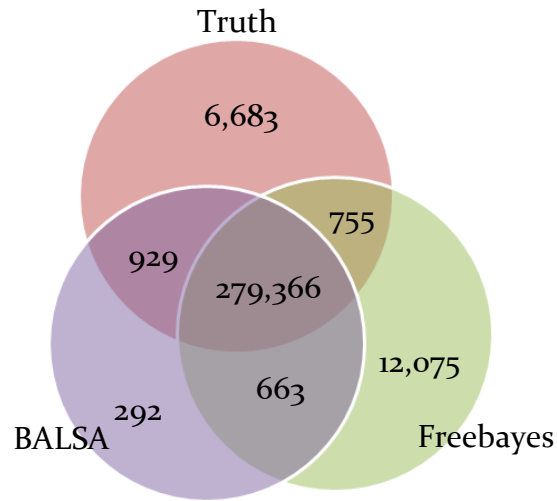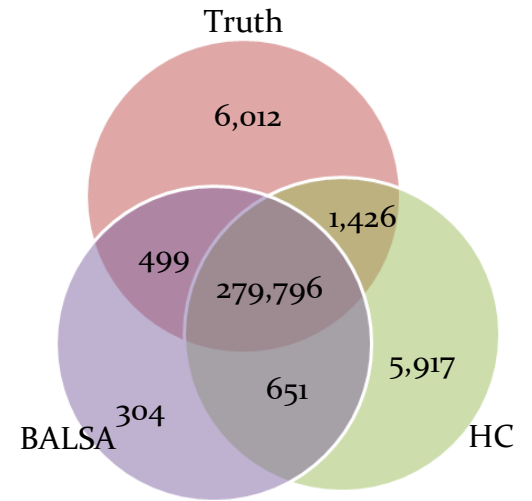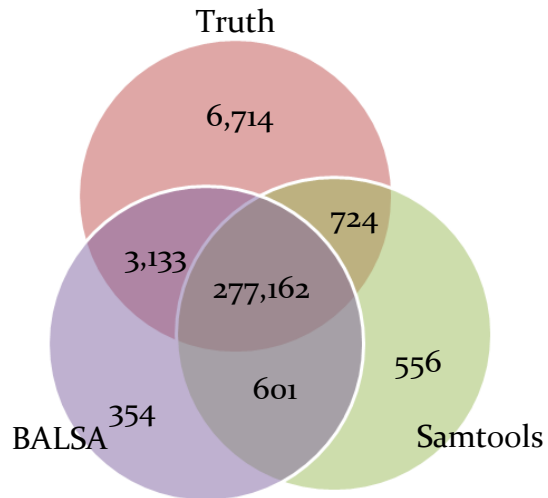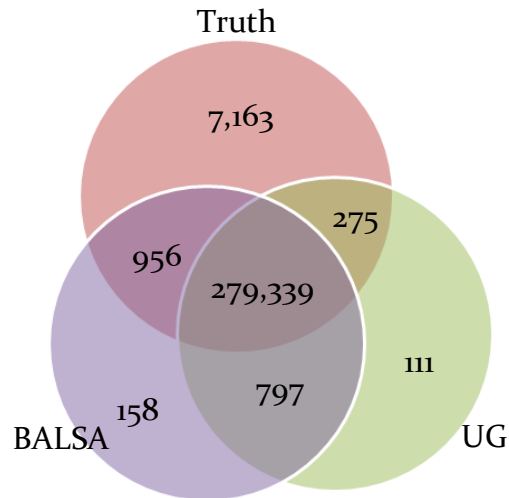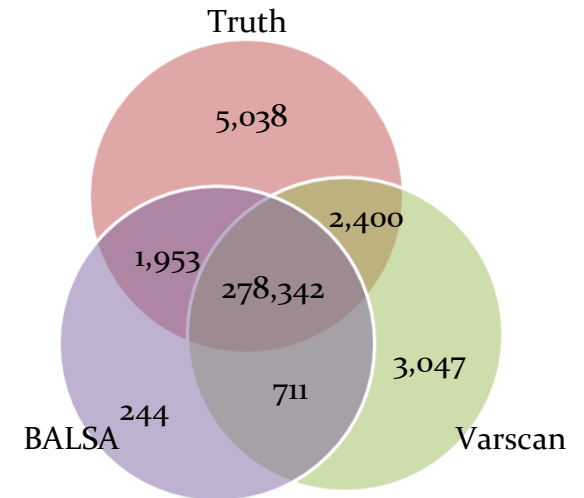

Supplement: Figure S2 [file peerj-02-421-s003.pdf]

# Size distribution of Indel

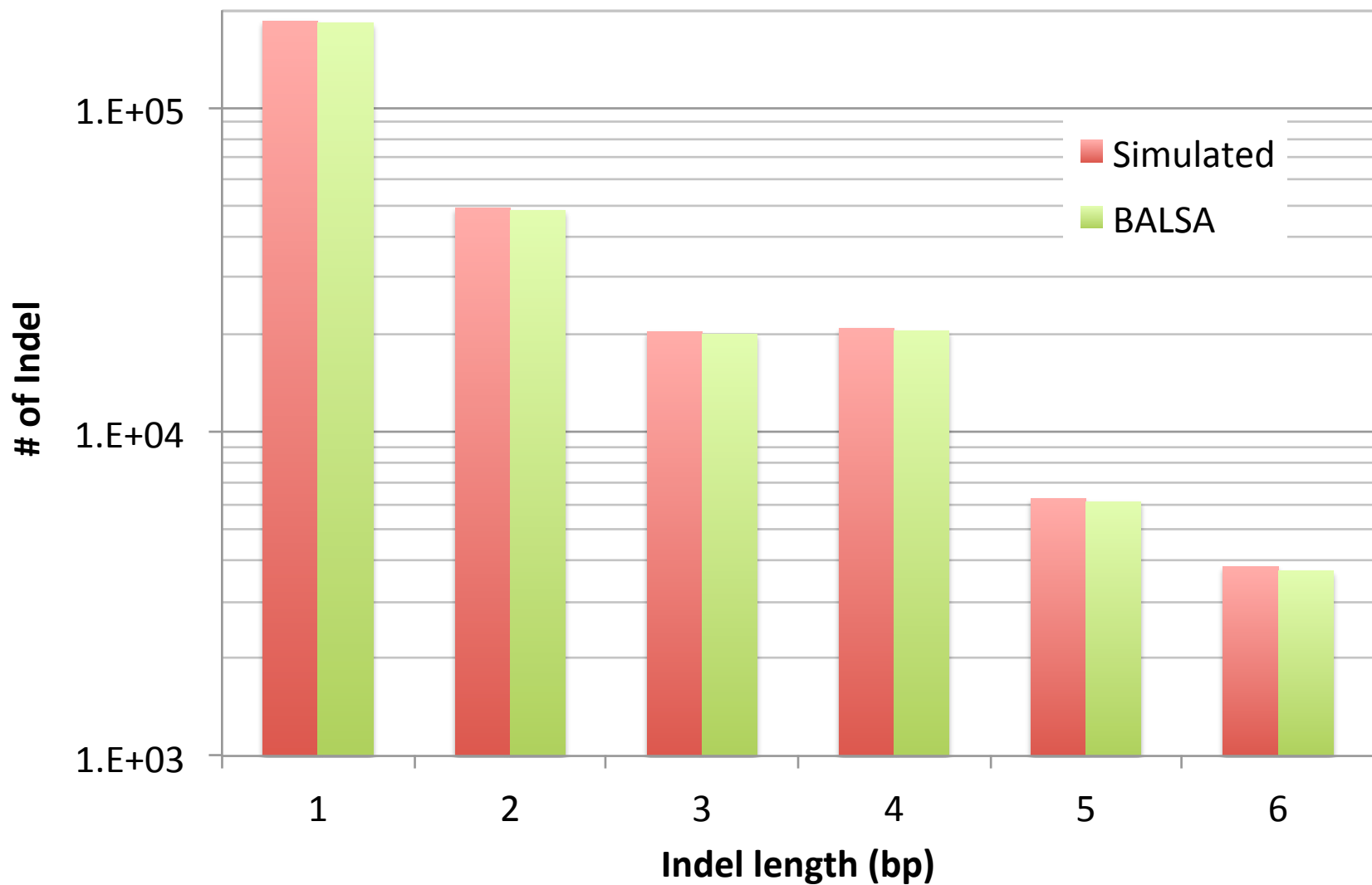

Supplement: Figure_S3 [file peerj-02-421-s004.pdf]
